# The influence of postoperative albumin levels on the outcome of cardiac surgery

**DOI:** 10.1186/s13019-020-01133-y

**Published:** 2020-05-11

**Authors:** David Berbel-Franco, Juan Carlos Lopez-Delgado, Alessandro Putzu, Francisco Esteve, Herminia Torrado, Elisabet Farrero, David Rodríguez-Castro, Maria Lluïsa Carrio, Giovanni Landoni

**Affiliations:** 1 Intensive Care Department, Hospital Universitari de Bellvitge, C/Feixa Llarga s/n. 08907, L’Hospitalet de Llobregat, Barcelona, Spain; 2 IDIBELL (Institut d’Investigació Biomèdica Bellvitge; Biomedical Investigation Institute of Bellvitge), Avda. Gran Via de L’Hospitalet 199, 08908, L’Hospitalet de Llobregat, Barcelona, Spain; 3 Department of Anesthesiology, Pharmacology & Intensive Care Medicine, Division of Anesthesiology, Geneva University Hospitals, Geneva, Switzerland; 4 Department of Anesthesia and Intensive Care, IRCCS San Raffaele Scientific Institute, Milan, Italy; 5 Vita-Salute San Raffaele University, Milan, Italy

**Keywords:** Cardiac surgery, Serum albumin levels, Perioperative medicine, Postoperative outcomes, In-hospital, Long-term mortality

## Abstract

**Background:**

The prognostic role of low postoperative serum albumin levels (SAL) after cardiac surgery (CS) remains unclear in patients with normal preoperative SAL. Our aim was to evaluate the influence of SAL on the outcome of CS.

**Methods:**

Prospective observational study. Patients undergoing CS with normal preoperative SAL and nutritional status were included and classified into different subgroups based on SAL at 24 h after CS. We assessed outcomes (i.e., in-hospital mortality, postoperative complications and long-term survival) and results were analyzed among the different subgroups of SAL.

**Results:**

We included 2818 patients. Mean age was 64.5 ± 11.6 years and body mass index 28.0 ± 4.3Kg·m^− 2^. 5.8%(*n* = 162) of the patients had normal SAL levels(≥35 g·L^− 1^), 32.8%(*n* = 924) low deficit (30–34.9 g·L^− 1^), 44.3%(*n* = 1249) moderate deficit (25–29.9 g·L^− 1^), and 17.1%(*n* = 483) severe deficit(< 25 g·L^− 1^). Higher SAL after CS was associated with reduced in-hospital (OR:0.84;95% CI:0.80–0.84; *P* = 0.007) and long-term mortality (HR:0.85;95% CI:0.82–0.87;*P* < 0.001). Subgroups of patients with lower SAL showed worst long-term survival (5-year mortality:94.3% normal subgroup, 87.4% low, 83.1% moderate and 72.4% severe;*P* < 0.001). Multivariable analysis showed higher in-hospital mortality, sepsis, hemorrhage related complications, and ICU stay in subgroups of patients with lower SAL. Predictors of moderate and severe hypoalbuminemia were preoperative chronic kidney disease, previous CS, and longer cardiopulmonary bypass time.

**Conclusions:**

The presence of postoperative hypoalbuminemia after CS is frequent and the degree of hypoalbuminemia may be associated with worst outcomes, even in the long-term scenario.

## Background

Liver proteins, such as albumin, have been considered over time as indicators of morbidity and mortality, as well as recovery markers of acute and chronic disease [1]. Serum levels may identify patients most likely to develop malnutrition or with a previous poor nutritional status, even when adequate nutritional supplementations during hospital stay is being performed [1]. Indeed, recent published guidelines suggest that surgery should be avoided in patients with low preoperative serum albumin levels (SAL) (< 30 g∙L^− 1^) due to its association with worst outcomes and impaired nutritional status [2].

Low preoperative SAL are associated with complications and mortality in the setting of cardiac surgery (CS) in patients who underwent coronary artery bypass graft (CABG), as well as other anthropometric values associated with nutritional status, such as body mass index (BMI) [3]. However, no correlation between low BMI (< 20 kg∙m^− 2^) and low SAL (< 25 g∙L^− 1^) has been reported [4]. Furthermore, SAL has been considered as an independent prognosis marker in several studies related with CS procedures, even in the setting of newer technology, such as transcatheter aortic valve replacement and left-ventricular assist device [5–7]. The level of hypoalbuminemia required to increase the risk of these outcomes has not been properly defined and varies depending on the study (i.e., under 20, 35 or 40 g∙L^− 1^) [8–10]. Increased risk of postoperative infection in patients with low preoperative SAL has also been reported [11].

The presence of low postoperative SAL after CS may be caused by several factors apart from low preoperative SAL, which include surgical injury, dilution due to fluid resuscitation, blood loss and cardiopulmonary bypass (CPB) among other factors [12]. The associated underlying pathophysiological mechanism is mainly related with the degree of ischemia-reperfusion injury or systemic inflammatory response syndrome (SIRS) produced during CS and a higher degree of SIRS is associated with higher risk of morbidities and mortality [13]. However, excessive fluid reanimation and blood loss can also influence in the occurrence of lower postoperative SAL [12]. Thus, postoperative hypoalbuminemia is the summation of different factors associated with outcomes after CS and postoperative SAL may have prognosis implications.

It is well known that the early identification of patients at risk for poor outcome after CS may be helpful in modifying patient care strategy in the Intensive Care Unit (ICU) in order to improve outcomes. A better understanding of the different aspects that contribute to morbidity and mortality is needed because CS population trend towards aging and an increasing incidence of comorbidities which ultimately affects the frailty of these patients [14]. In addition, measuring long-term outcomes is a marker of the quality of perioperative care in CS [15]. Monitoring of postoperative SAL may be a helpful tool for risk stratification and prognosis assessment due to the different cumulative aspects that SAL entails (e.g., inflammatory response, fluid status and malnutrition) [12]. The aim of the present study was to evaluate the influence of postoperative SAL on the short- and long-term outcomes of patients who underwent CS with normal preoperative SAL. We also aimed to identify those factors related with postoperative moderate to severe hypoalbuminemia (< 30 g∙L^− 1^).

## Methods

A prospective observational study was performed in a surgical Intensive Care Unit (ICU) of a university affiliated referral hospital between June 2006 and September 2011. All consecutive patients undergoing different types of CS with preoperative normal albumin levels (i.e., SAL > 35 g∙L^− 1^) and appropriate nutritional status (evaluated by means of Subjective Global Assessment) were considered for the study. Heart-transplant patients were excluded due to the potential influence of immunosuppressant and/or corticosteroid therapy over postoperative SAL and inflammatory response (see Supplementary Figure 1). The study was approved by the Institutional Ethics Committee of our hospital (Comité d’Ètica i Assajos Clínics de Hospital Universitari de Bellvitge; Barcelona, Spain); with approval number 39/07. Informed consent was waived due to the observational nature of our study (Details of the ethics approval and study protocol are provided as supplementary material).

Data were prospectively extracted from the medical registry of each patient and collected in a local database for analysis purposes. Preoperative data (demographic data, comorbidities and treatment before surgery), operative data and postoperative variables were routinely collected together with CS scores (Parsonnet, European System for Cardiac Operative Risk Evaluation (EuroSCORE)) and ICU scores (Acute Physiology and Chronic Health Evaluation (APACHE) II and III, Simplified Acute Physiology Score (SAPS) II and III). A follow-up during 4.6 ± 2.4 years was performed in 2565 patients (Follow-up was not possible in 94 patients that were alive at hospital discharge). The long-term follow-up was performed using the Catalan Health Central Registry (*Registre Central de Persones Assegurades*).

Postoperative SAL was measured 24 h after CS and patients were classified into different categories based on local laboratory categories of SAL and previous research [4, 6]: normal (≥35 g∙L^− 1^) (*n* = 162), low deficit (30–34.9 g∙L^− 1^) (*n* = 924), mild deficit (25–29.9 g∙L^− 1^) (*n* = 1249) and severe deficit (< 25 g∙L^− 1^) (*n* = 483). We chose 24 h postoperative for measuring SAL because based on our clinical experience unstable patients received usually major part of fluid resuscitation and blood products from ICU admission to 24 h, which may alter SAL the most, and because 24 h is the timeline used in ICU for prognosis assessment [16].

Recent acute myocardial infarction (AMI) was defined as an AMI that required admission to the hospital during the last month before surgery or an AMI that did not allow discharge from the hospital before surgery. The other definitions used for this study were based on the Society of Thoracic Surgeons’ national CS database definitions [17].

The operations were performed by the same group of surgeons during the study period following standards of practice. The priming fluid for the CPB system were crystalloids during the study period with a priming volume of the circuit between 500 and 800 mL. In all patients, decisions regarding perioperative management were made by the attending physician according to local protocols. Patients were treated according to hemodynamic parameters and metabolic markers of tissue perfusion, such as arterial lactate levels and venous oxygen saturation. Fluid resuscitation was performed based on local protocol following a restricted fluid regimen in order to avoid excessive positive fluid balance (i.e., > 2 L of positive fluid balance per day) [18]. Our hemodynamic objectives were to achieve both appropriate mean arterial pressure (MAP) (i.e., about 70 mmHg or the previously reported usual MAP in each patient) and urine output (i.e., > 0.5 mL·Kg^− 1^·h^− 1^ or higher to avoid positive fluid balance). Global end-diastolic volume index (about 680–800 mL·m^− 2^) or cardiac index (about 2.5 L·min^− 1^·m^− 2^) were also monitored in the presence of high dose of vasopressors or inotropes [19]. We monitored central venous pressure to see the dynamic changes over time and fluid responsiveness to passive leg raise in conjunction with previous clinical evaluation for hemodynamic management [19]. Fluid loading was performed by crystalloids. According with our previous research, our perioperative transfusion trigger is hemoglobin value between 7 and 8 g·dL^− 1^ with a targeted hematocrit on CPB about 21–24% [20]. Transfusions occurring during fist 24 h were assessed due to the potential influence over SAL. Supplemental albumin administration was not specifically forbidden but none was administered to any patient during the study period. A glycemic protocol was applied during and after CS based on local protocols in order to avoid blood glucose levels > 180 mg·dL^− 1^ during and/or after CS.

### Statistical analyses

Statistical analysis was conducted using PASW statistics 20.0 (SPSS Inc., Chicago, Illinois, USA). Data are expressed as mean ± standard deviation or median (interquartile range) as appropriate. In order to evaluate the association of postoperative SAL with mortality after CS, we analyzed differences between survivors and non-survivors with univariate analyses (for comparisons between groups the Mann-Whitney U test was used or, when appropriate, the two-sample t-test; the χ^2^-test was used to evaluate categorical prognostic factors) to identify the importance of postoperative SAL as a factor associated with mortality. ANOVA was used to compare differences in characteristics and outcome between different hypoalbuminemia subgroups (*P* values shown in tables) and subsequent post hoc tests (Bonferroni tests) were used to determine and confirm significant differences in the various pairwise comparisons (*P* values shown in results section).

Multivariate analysis was carried out using a backward stepwise logistic regression to identify predictors of in-hospital mortality after CS. Variables with *P* < 0.1 were included in the initial model and according the criteria of the investigators (i.e., redundant and associated variables were avoided). Change-in-estimates criterion and backwards deletion with a 10% cutoff was used to eliminate confounding variables from our final models. Proportional Hazards Cox regression model was used to evaluate predictors of long-term mortality after adjusting for the time of follow-up period.

We tested for interactions between the variables that we introduced into all the multivariate analyses, in order to avoid destabilization of the different analyses. We performed adjustment for age, preoperative albumin, type of cardiac surgery, CPB time, EuroSCORE and the significant preoperative differences between the subgroups in order to avoid the influence of the severity of illness when outcomes were analyzed. Finally, we also analyze using the same statistical methods preoperative and intraoperative variables in order to show factors associated with the occurrence of postoperative moderate to severe hypoalbuminemia.

In all cases, the Kolmogorov-Smirnov test and D’Agostino-Pearson omnibus normality test were used to check the normal distribution of our population and to assess the goodness-of-fit of the final regression models. Survival analysis was carried out using the Kaplan-Meier estimator for the different hypoalbuminemia subgroups. Proportional Hazards Cox regression model was used to confirm survival analysis, which was risk-adjusted in order to avoid the severity of illness. Two-tailed *P* value < 0.05 was considered statistically significant.

## Results

### Baseline characteristics and postoperative SAL as a factor associated with mortality and survival

Patients were 2818 and overall they had a hospital mortality of 5.6%. Characteristics of our studied population are provided in Table 1. Preoperative, intraoperative and postoperative data comparing survivors and non-survivors showed that non-survivors were older, underwent longer CPB during CS, had lower SAL and higher rates of morbidities and postoperative complications (see Table 1). Multivariate analysis showed that lower levels of SAL (i.e., SAL as absolute value) were both associated with increased in-hospital (Odds ratio (OR): 0.844, 95% confidence interval (CI) 0.805 to 0.844, *P* = 0.007) and long-term mortality (Hazard ratio (HR): 0.846, 95% CI 0.821 to 0.871, *P* < 0.001). Other variables, such as older age, having a dilated cardiomyopathy in the preoperative echocardiography, suffering a CPB > 100 min, suffering from postoperative acute renal failure, low cardiac output syndrome and sepsis during the postoperative period, were also associated with mortality (Table 2; see Supplementary Table 1 for full model results).

**Table 1:** Preoperative (A), intraoperative and postoperative (B) characteristics of the population

**A**	All patients (*n* = 2818)	Hospital Survivors (*n* = 2659; 94.4%)	Non-survivors (*n* = 159; 5.6%)	*P*
**Preoperative data**				
Age (years)	64.5 ± 11.6	64.2 ± 11.6	69.7 ± 10	***< 0.001***
Sex (male)	63.8% (1799)	63.7% (1696)	64.7%(103)	*0.86*
BMI (Kg·m^−2^)	28.0 ± 4.3	27.9 ± 4.3	28.0 ± 4.6	*0.89*
Albumin before surgery (g ·L^−1^)	40.0 ± 3.5	40.1 ± 2.8	39.9 ± 1.5	*0.85*
Subjective Global Assessment (class B)	5.8% (163)	5.9% (158)	3.1% (5)	*0.25*
Hypertension	62.7% (1768)	62.1% (1650)	74.2%(118)	***0.002***
Diabetes Mellitus	24.8% (699)	24.9% (662)	33.3% (53)	***0.03***
Dyslipidemia	50.5% (1423)	50.4%(1339)	52.8% (84)	*0.56*
Peripheral vascular disease	8.8% (249)	8.3% (220)	18.2% (29)	***< 0.001***
Chronic renal insufficiency	5.2% (146)	4.6% (122)	15.1%(24)	***< 0.001***
Renal failure (on Dialysis)	0.8% (23)	0.8% (20)	1.9%(3)	*0.14*
Creatinine before surgery (μmol·l^−1^)	96 ± 59	94 ± 58	119 ± 67	***< 0.001***
Previous stroke	5.6% (158)	5.5% (145)	8.2% (13)	*0.15*
COPD	12% (337)	11.5%(307)	18.9%(30)	***0.008***
Active smokers	15.5% (437)	15.3%(407)	18.8%(30)	*0.58*
Previous atrial fibrillation	23.8% (671)	23.3%(620)	32.8%(51)	***0.005***
Previous myocardial infarction	15.5% (437)	15.3% (407)	18.9% (30)	*0.26*
Recent myocardial infarction	11% (310)	10.5% (278)	20.1% (32)	***0.001***
On B-Blockers	41.3% (1165)	41.4% (1102)	39.6% (63)	*0.68*
On statins	41.2% (1160)	41.3% (1097)	39.6% (63)	*0.74*
On Aspirin	44.5% (1184)	44.5% (1184)	43.4% (69)	*0.81*
On diuretics	47.6% (1340)	46.6% (1239)	63.5% (101)	***0.001***
Hypertrophic cardiomyopathy	31.2% (880)	31% (826)	33.9% (54)	*0.59*
Dilated cardiomyopathy	20.4% (577)	20.2% (537)	25.1% (40)	*0.19*
LVEF (%)	60 ± 12	59 ± 13	60 ± 12	*0.25*
PAP (mmHg)	46 ± 16	45 ± 15	49 ± 16	***0.04***
Hemoglobin before surgery (g·dL^**−1**^)	13.0 ± 1.7	13.0 ± 1.7	12.0 ± 1.9	***0.001***
Platelet count before surgery (1·nl^**− 1**^)	215 ± 68	216 ± 68	208 ± 75	*0.24*
Emergent surgery	5.1% (143)	4.5% (120)	14.5% (23)	***< 0.001***
Past cardiac surgery	9.4% (266)	9.2% (245)	13.2% (21)	*0.09*
EuroSCORE	5.9 ± 3	5.7 ± 2.8	8.6 ± 3.8	***< 0.001***
Parsonnet score	11.4 ± 7.4	11.2 ± 7.2	15.4 ± 9.8	***< 0.001***
**B**	All patients (n = 2818)	Hospital Survivors (n = 2659; 94.4%)	Non-survivors (n = 159; 5.6%)	*P*
**Intraoperative data**				
Isolated CABG	32.2% (907)	32.3% (859)	30.2% (48)	*0.86*
Isolated valve surgery	51.7% (1456)	52% (1382)	46.5% (74)	*0.65*
CABG + valve surgery	6.9% (194)	6.7% (178)	10.1% (16)	*0.09*
Other cardiac surgery	9.2% (261)	9% (240)	13.2% (21)	*0.06*
Number of bypass	3 (2–4)	3 (2–4)	3 (2–4)	*0.85*
CPB time (min)	112 ± 41	111 ± 39	140 ± 62	***0.001***
ACC time (min)	74 ± 30	73 ± 29	84 ± 37	***0.001***
**Postoperative data**				
APACHE II	12.3 ± 4.6	11.8 ± 4.1	19 ± 6.8	***< 0.001***
APACHE III	50 ± 18.3	48.2 ± 16	78.1 ± 27.8	***< 0.001***
SAPS II	24 ± 9.6	23.3 ± 8.4	38 ± 14.6	***< 0.001***
SAPS III	40.0 ± 10.4	39.3 ± 9.6	51.8 ± 13.5	***< 0.001***
Ventilation time (hours)	51 ± 129	39 ± 99	248 ± 309	***< 0.001***
PaO2/FiO2 ratio on admission	331 ± 98	333 ± 97	290 ± 112	***< 0.001***
PaO2/FiO2 ratio 12 h after admission	311 ± 89	315 ± 87	249 ± 98	***< 0.001***
PaO2/FiO2 ratio 24 h after admission	308 ± 76	312 ± 73	236 ± 92	***< 0.001***
Reintubation	1.1% (31)	1% (25)	3.8% (6)	***0.01***
Tracheostomy	1.3% (35)	1% (26)	5.7% (9)	***0.005***
Need of vasoactive drugs (hours)	103 ± 141	91 ± 116	253 ± 271	***< 0.001***
LCOS	41.5% (1170)	38.8% (1034)	85.5% (136)	***< 0.001***
PMI	11.6% (327)	10.2% (272)	34.6% (55)	***< 0.001***
IABP support	7.8% (222)	6.7% (180)	26.4% (42)	***< 0.001***
Atrial Fibrilation	39.5% (1114)	37.9% (1009)	66% (105)	***< 0.001***
Albumin 24 h after surgery (g ·L^−1^)	28 ± 4.8	28 ± 2.4	25 ± 3.5	***< 0.001***
AL peak after surgery (mmol·l^−1^)	3.8 ± 1.8	3.6 ± 1.4	5.9 ± 4.3	***< 0.001***
Acute Renal Failure	9.7% (272)	6.5% (174)	61.6% (98)	***< 0.001***
Need for RRT	2.1% (58)	0.8% (22)	22.6% (36)	***< 0.001***
Haemorrhage-related reexploration	3.4% (97)	3% (81)	10.1% (16)	***< 0.001***
Pericardial tamponade	0.7% (19)	0.6% (17)	1.3% (2)	*0.29*
Drainage loss first 12 h (ml)	392 ± 295	386 ± 287	492 ± 398	***< 0.001***
Re-exploration	1.6% (47)	1.1% (30)	10.7% (17)	***< 0.001***
Need for blood products first 24 h (Units)	1.2 ± 1.9	1.1 ± 1.6	3.2 ± 3.9	***0.01***
Stroke	1.4% (39)	1.1% (28)	6.9% (11)	***< 0.001***
Septicaemia	6.6% (186)	4.6% (122)	40.2% (65)	***< 0.001***
Mean ICU stay (hours)	125 ± 158	114 ± 131	301 ± 314	***0.003***
Mean hospital stay (days)	25.0 ± 20.3	22.3 ± 18.3	36.4 ± 58.2	***< 0.001***

*BMI* Body Mass Index, COPD Chronic Obstructive Pulmonary Disease, *NYHA* New York Heart Association classification, *LVEF* Left ventricular ejection fraction, *PAP* Pulmonary arterial pressure, *EuroSCORE* European system for cardiac operative risk evaluation, *CABG* coronary artery bypass graft, *CPB* Cardiopulmonary Bypass, *ACC* Aortic cross clamping, *APACHE* Acute Physiology and Chronic Health Evaluation, *SAPS* Simplified Acute Physiology Score, *PaO2/FiO2* Arterial partial pressure of O2 and fraction of inspired oxygen ratio, *LCOS* Low Cardiac Output Syndrome, *PMI* Perioperative Myocardial Infarction, *IABP* intra-aortic balloon pump, *AL* Arterial Lactate, *RRT* Renal Replacement Therapy. Results are expressed as mean ± standard deviation, percentage or median and interquartile range

**Table 2:** Multivariate analysis – variables associated with in-hospital and long-term mortality

**Dependent variable in-hospital mortality**	Odds ratio (95% Confidence Interval)	*P*-value
Age	1.050 (1.027–1.075)	***< 0.001***
Cardiopulmonary bypass time (> 100 min)	1.007 (1.003–1.010)	***0.001***
Albumin 24 h after surgery (g·L^−1^)	0.844 (0.805–0.844)	***0.007***
**Dependent variable long-term mortality**	Hazards ratio (95% Confidence Interval)	*P*-value
Age	1.063 (1.049–1.076)	***< 0.001***
Cardiopulmonary bypass time (> 100 min)	1.004 (1.002–1.006)	***0.001***
Dilated cardiomyopathy	1.435 (1.139–1.810)	***0.002***
Albumin 24 h after surgery (g·L^−1^)	0.846 (0.821–0.871)	***< 0.001***
Acute Renal Failure	2.523 (1.395–2.933)	***< 0.001***
Low Cardiac Output Syndrome	1.489 (1.276–1.656)	***0.006***
Septicaemia	1.125 (1.018–1.696)	***0.02***

**Fig. 1:**
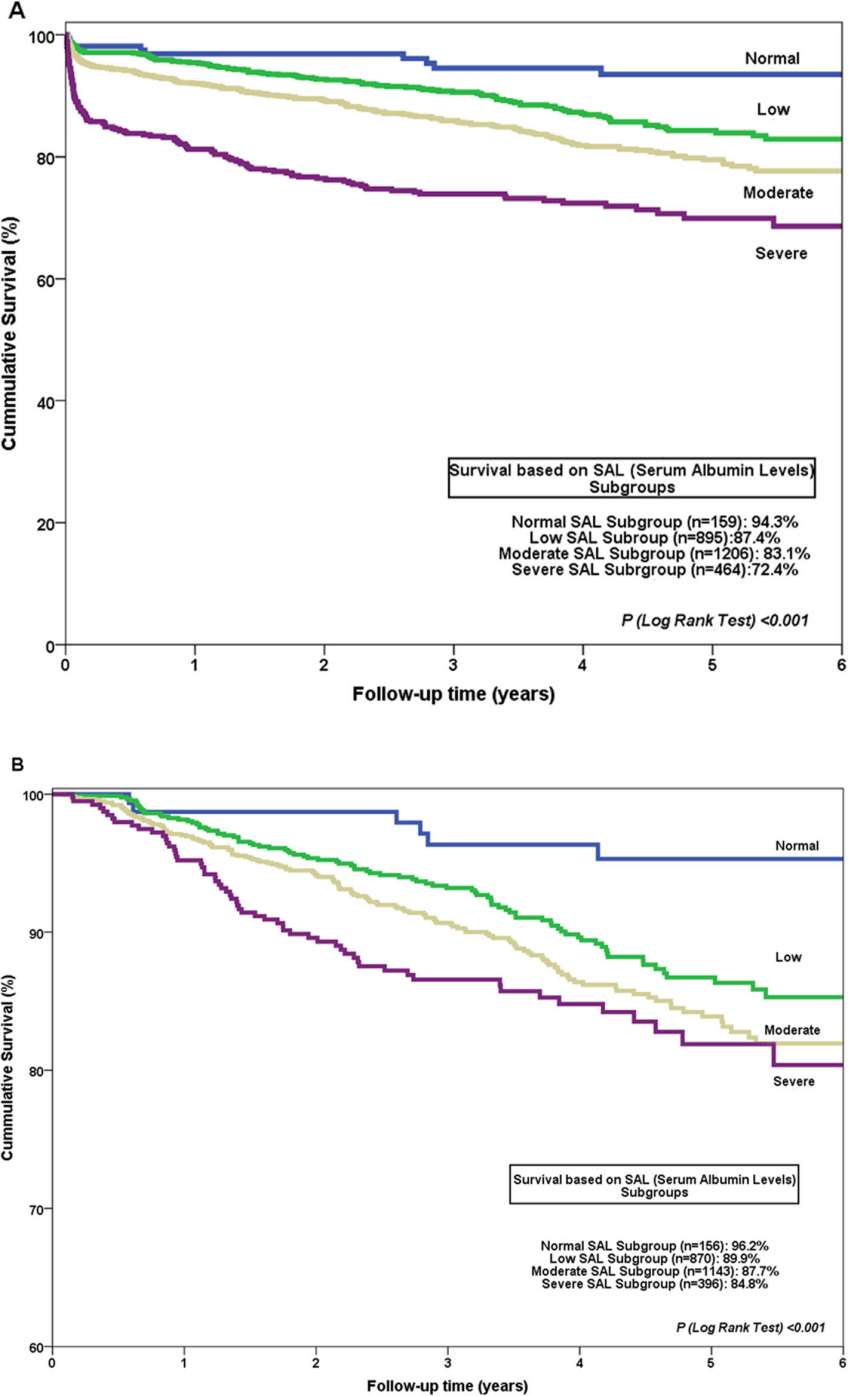
Kaplan-Meier survival curves based on subgroups of patients with different albumin levels 24 h after cardiac surgery including in-hospital mortality (**a**) and only with hospital survivors (**b**)

**Fig. 2:**
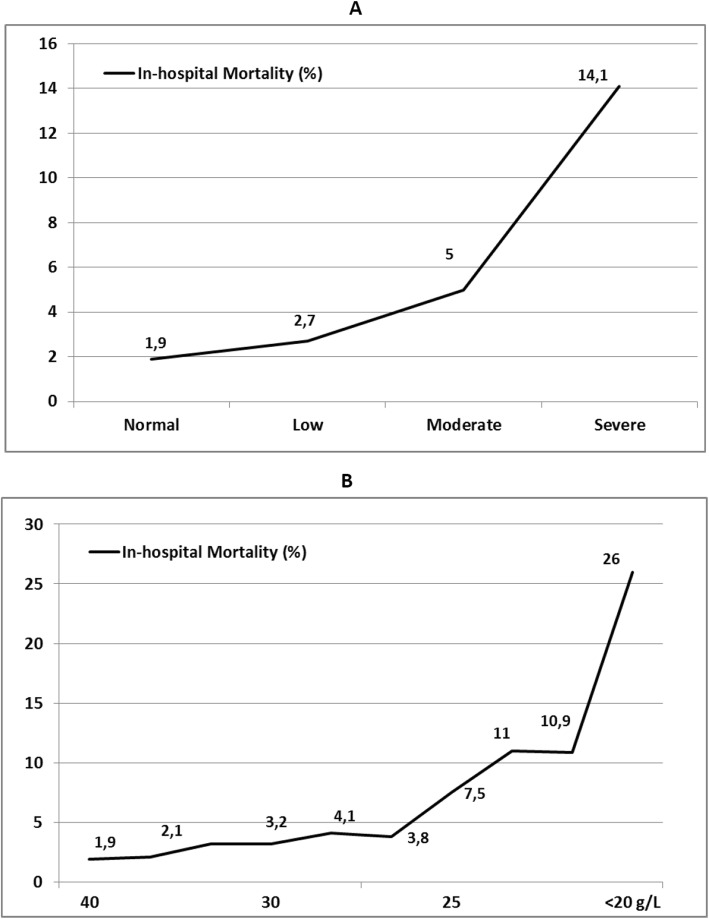
Relationship between in-hospital mortality rates and postoperative Serum Albumin Levels based on subgroups (**a**) and degree of hypoalbuminemia (**b**)

A follow-up was performed in all patients for in-hospital mortality in 2659 patients with a mean follow-up of 4.6 ± 2.4 years. There was shown a lower survival rate and higher mortality over time in patients with lower SAL, and particularly in the severe albumin deficit group (Fig. 1 and Table 3). Additional analyses showed that normal SAL (≥35 g∙L^− 1^) was associated with better in-hospital survival (HR = 0.570, 95% CI 0.388 to 0.836, *P* = 0.004) and long term survival (HR = 0.315, 95% CI 0.145 to 0.682, *P* = 0.003) whereas moderate (25–29.9 g∙L^− 1^) (HR = 1.604, 95% CI 1.452 to 1.806, *P* = 0.001) and severe deficits (< 25 g∙L^− 1^) (HR = 1.966, 95% CI 1.520 to 2.853, *P* = 0.001) were independent risk factors for increased long-term mortality. The relationship between postoperative SAL and in-hospital mortality was not linear and progressively higher among subgroups depending on the severity of hypoalbuminemia, as showed by Fig. 2.

**Table 3:** Long-term mortality based on subgroups of patients with different albumin levels 24 h after cardiac surgery including all patients (A) and only with hospital survivors (B)

**A**	In-hospital survival	1-year survival	3-year survival	5-year survival
Normal Levels (≥35 g·L^−1^)	98.1%	98.1%	96.5%	94.3%
Low deficit (30–34.9 g·L^−1^)	97.3%	94.2%	89.2%	87.4%
Mild deficit (25–29.9 g·L^−1^)	95%	91.5%	86.1%	83.1%
Severe deficit (< 25 g·L^−1^)	85.9%	78.3%	74.5%	72.4%
**B**				
Normal Levels (≥35 g·L^−1^)	100%	98.9%	97.8%	96.2%
Low deficit (30–34.9 g·L^−1^)	100%	97.9%	94.2%	89.9%
Mild deficit (25–29.9 g·L^−1^)	100%	96.8%	92.3%	87.7%
Severe deficit (< 25 g·L^−1^)	100%	95.1%	87.5%	84.8%

### Differences between postoperative SAL subgroups and risk factors for hypoalbuminemia

When we compared between postoperative SAL subgroups (*P* from ANOVA test provided in Table 4), preoperative variables showed older patients, higher cardiovascular risk factors (such as hypertension, dyslipidemia and diabetes mellitus) and an increased prevalence of chronic renal insufficiency in low SAL determination groups (Bonferroni post hoc test; *P* < 0.001). In addition, there was subgroup differences in hemoglobin determinations before surgery (*P* = 0.001). Patients with severe SAL deficit required longer CPB and aortic cross clamping (ACC) times compared with the other groups (*P* < 0.001). Postoperative variables showed longer ventilation time and vasoactive drug requirements for low SAL subgroups (*P* < 0.001) and a higher acute renal failure incidence in the severe SAL deficit group when compared with other subgroups (*P* < 0.001). Mean ICU and hospital stay was higher for the low SAL determination groups, especially in the severe deficit group (*P* < 0.001). In-hospital mortality was also higher in this group when compared to others (*P* < 0.001).

**Table 4:** Preoperative (A), intraoperative and postoperative (B) variables of subgroups of patients with different albumin levels 24 h after cardiac surgery

**A**	Normal Levels (≥35 g ·L^−1^) n = 162 5.8%	Low deficit (30–34.9 g ·L^− 1^) n = 924 32.8%	Mild deficit (25–29.9 g ·L^− 1^) n = 1249 44.3%	Severe deficit (< 25 g ·L^− 1^) n = 483 17.1%	*ANOVA* *P value*
Sex (male)	75% (122)	68% (634)	61% (765)	57% (278)	***0.001***
Age (years)	57 ± 14.1	63 ± 11.6	65.5 ± 11.2	67.7 ± 10	***< 0.001***
Albumin before surgery (g ·L-1)	41 ± 2.5	41 ± 2.8	39 ± 2.9	39 ± 2.2	*0.78*
Subjective Global Assessment (class B)	6.1% (10)	5.9% (55)	58.8% (73)	5.1% (25)	*0.89*
Hypertension	53.0% (86)	60.4% (558)	63.4% (792)	68.7% (332)	***0.001***
Dyslipidemia	42.0% (68)	47.6% (440)	53.1% (663)	52.2% (252)	***0.009***
Diabetes Mellitus	19.1% (31)	24.3% (225)	26.1% (327)	27.1% (131)	*0.06*
BMI (kg·m^−2^)	27.6 ± 4.6	28.3 ± 4.2	27.9 ± 4.2	27.4 ± 4.3	*0.90*
Peripheral vascular disease	3.7% (6)	6.8% (63)	10.2% (127)	11% (53)	***0.001***
Chronic renal insufficiency	2.5% (4)	2.9% (27)	5.2% (65)	10.4% (50)	***< 0.001***
Renal Failure (on Dialysis)	0.6% (1)	0.4% (4)	0.7% (9)	1.9% (9)	*0.04*
Creatinine before surgery (μmol·l^−1^)	86 ± 36	88 ± 34	96 ± 61	110 ± 84	***0.001***
Previous Stroke	2.5% (4)	5.4% (50)	5.4% (68)	7.5% (36)	*0.99*
COPD	8% (13)	11.8% (109)	12.4% (155)	12.4% (60)	*0.43*
Active smokers	19.1% (31)	15.9% (147)	14.9% (187)	14.9% (72)	*0.42*
Previous Atrial Fibrillation	25.9% (42)	24% (222)	23% (288)	24.6% (119)	*0.59*
Previous Myocardial Infarction	14.2% (23)	14.7% (136)	16% (200)	16.1% (78)	*0.79*
Recent Myocardial Infarction	4.9% (8)	7.7% (71)	11.8% (148)	17.2% (83)	***0.001***
On B-Blockers	34.6% (56)	41.2% (381)	41.6% (520)	43.1% (208)	*0.82*
On statins	30.2% (49)	40.3% (372)	43.3% (541)	41% (198)	***0.01***
On Aspirin	28.4% (46)	42.2% (390)	47.6% (594)	46.2% (223)	***0.001***
On diuretics	47.5% (77)	45.1% (417)	48.7% (608)	49.3% (238)	*0.33*
Hypertrophic cardiomyopathy	27.1% (44)	31.6% (292)	32.9% (412)	27.3% (132)	*0.11*
Dilated cardiomyopathy	27.1% (44)	23% (213)	19.3% (242)	16.1% (78)	***0.02***
LVEF (%)	60 (48–72)	61 (47–75)	60 (41–79)	60 (43–77)	*0.34*
PAP (mmHg)	48 (37–59)	32 (21–43)	37 (21–53)	35 (18–52)	*0.26*
Hemoglobin before surgery (g·dL^**−1**^)	13.5 ± 1.5	13.3 ± 1.6	12.9 ± 1.7	12.4 ± 1.8	***0.001***
Platelet count before surgery (1·nL^**− 1**^)	203 ± 49	213 ± 67	215 ± 68	224 ± 75	***0.002***
Past Cardiac surgery	12.3% (20)	9.4% (87)	9.1% (114)	9.3% (45)	*0.62*
Emergent Surgery	0.6% (1)	0.9% (8)	4.6% (58)	15.7% (76)	***< 0.001***
EuroSCORE	5.2 ± 3.2	5.7 ± 2.8	6.2 ± 1.9	7.3 ± 3.2	*0.14*
Parsonnet score	11.0 ± 6.2	11.1 ± 5.2	12.8 ± 3.7	13.4 ± 2.8	*0.25*
Mean Pre-ICU stay (days)	1.1 ± 0.3	1.1 ± 0.4	1.0 ± 0.3	1.9 ± 2.1	***0.02***
**B**	Normal Levels (≥35 g ·L^−1^) n = 162 5.8%	Low deficit (30–34.9 g ·L^− 1^) n = 924 32.8%	Mild deficit (25–29.9 g ·L^− 1^) n = 1249 44.3%	Severe deficit (< 25 g ·L^− 1^) n = 483 17.1%	*ANOVA* *P value*
**Intraoperative data**					
Isolated CABG	20.4% (33)	32.3% (298)	32.5% (406)	35.2% (170)	***0.001***
Isolated valve surgery	64.2% (104)	56.5% (522)	50% (624)	42.7% (206)	***0.001***
CABG + valve surgery	3.1% (5)	4.9% (45)	8.6% (107)	7.7% (37)	***0.001***
Other cardiac surgery	12.3% (20)	6.4% (59)	9% (112)	14.5% (70)	***0.001***
Number of bypass	2 (2–3)	2 (2–3)	2 (2–3)	2 (2–4)	*0.85*
CPB time (min)	95 ± 39	105 ± 35	114 ± 41	127 ± 47	***< 0.001***
ACC time (min)	65 ± 30	69 ± 27	75 ± 29	81 ± 32	***< 0.001***
**Postoperative data**					
APACHE II	12.2 ± 2.2	11.9 ± 3.1	12.5 ± 5.8	12.9 ± 4.3	*0.15*
APACHE III	53.1 ± 17.9	49.2 ± 11.5	68.1 ± 20.6	75.9 ± 26.8	***< 0.001***
SAPS II	24 ± 8.6	22.3 ± 8.2	25.8 ± 12.4	28.2 ± 14.6	***0.01***
SAPS III	39.5 ± 8.4	38.2 ± 8.5	43.2 ± 10.5	45.3 ± 12.6	***< 0.001***
Ventilation time (hours)	6 (4–8)	19.5 (11.5–30.5)	18 (0–72)	21 (0–242)	***< 0.001***
PaO2/FiO2 ratio on admission	323 ± 91	330 ± 95	336 ± 96	319 ± 110	*0.21*
PaO2/FiO2 ratio 12 h after admission	338 ± 87	319 ± 83	310 ± 90	290 ± 95	***< 0.001***
PaO2/FiO2 ratio 24 h after admission	328 ± 72	318 ± 67	306 ± 76	284 ± 89	***< 0.001***
Reintubation	0	0.5% (5)	1.6% (20)	1.2% (6)	*0.10*
Tracheostomy	0.6% (1)	0.6% (6)	1.1% (14)	2.8% (14)	*0.09*
AL peak after surgery (mmol·l^−1^)	3.5 ± 1.3	3.7 ± 1.7	3.7 ± 1.7	4.2 ± 2.3	***0.001***
Need of vasoactive drugs (hours)	67 ± 92	75 ± 112	101 ± 139	152 ± 173	***< 0.001***
LCOS	17.3% (28)	28.3% (262)	44.9% (562)	65.8% (318)	***< 0.001***
PMI	8% (13)	9.1% (84)	11.8% (147)	17.2% (83)	***< 0.001***
IABP support	0.6% (1)	4.3% (40)	7.4% (93)	18.2% (88)	***0.001***
Atrial Fibrillation	33.3% (54)	34.4% (318)	40.3% (503)	49.5% (239)	***0.001***
Acute Renal Failure	3.1% (5)	4.4% (41)	9.7% (121)	21.7% (105)	***< 0.001***
Need for RRT	0	0.4% (4)	1.8% (22)	6.6% (32)	***< 0.001***
Haemorrhage-related reexploration	0	1.3% (12)	4.2% (52)	6.8% (33)	***0.001***
Pericardial tamponade	0	0.2% (2)	0.9% (11)	1.2% (6)	*0.07*
Drainage loss first 12 h (ml)	307 ± 163	355 ± 255	399 ± 298	472 ± 369	*0.33*
Re-exploration	1.2% (2)	0.5% (5)	1.8% (23)	3.2% (17)	*0.07*
Need for blood products first 24 h (Units)	1 (0–2)	1 (0–6)	1 (0–3)	1 (0–4)	*0.08*
Stroke	0.6% (1)	1% (9)	1.8% (22)	1.4% (7)	*0.38*
Septicaemia	2.4% (4)	3.1% (29)	6.4% (81)	14.9% (72)	***< 0.001***
Mean ICU stay (hours)	158 ± 213	190 ± 270	274 ± 315	327 ± 377	***0.001***
Mean hospital stay (days)	18 ± 11	21 ± 14	26 ± 21	33 ± 30	***0.001***
In-hospital mortality	1.9% (3)	2.7% (25)	5% (63)	14.1% (68)	***< 0.001***

*BMI* Body Mass Index, *COPD* Chronic Obstructive Pulmonary Disease, *NYHA* New York Heart Association classification, *LVEF* Left ventricular ejection fraction, *PAP* Pulmonary arterial pressure; *EuroSCORE* European system for cardiac operative risk evaluation, *CABG* coronary artery bypass graft; *CPB* cardiopulmonary bypass, *ACC* Aortic cross clamping, *APACHE* Acute Physiology and Chronic Health Evaluation, *SAPS* Simplified Acute Physiology Score, *PaO2/FiO2* Arterial partial pressure of O2 and fraction of inspired oxygen ratio, *LCOS* Low Cardiac Output Syndrome, *PMI* Perioperative Myocardial Infarction, *IABP* intra-aortic balloon pump, *AL* Arterial Lactate, *RRT* Renal Replacement Therapy. Results are expressed as mean ± standard deviation, percentage or median and interquartile range

All these findings regarding the differences in outcomes between the SAL subgroups from the univariate analysis were analyzed by means of multivariate analysis. A higher mortality rate, longer ICU stay and higher incidence of bleeding and sepsis-related complications were associated with lower levels of postoperative SAL when we made comparisons between the different postoperative SAL subgroups (Table 5; see Supplementary Table 2 for full model results).

**Table 5:** Differences between subgroups of patients with different albumin levels 24 h after cardiac surgery

	Odds ratio (95% CI)	***P value***
**Normal Levels** (≥35 g·L^−1^) **vs Low deficit** (30–34.9 g·L^−1^)		
In-hospital mortality	1.018 (1.002–1.034)	***0.02***
**Normal Levels** (≥35 g·L^− 1^) **vs Mild deficit** (25–29.9 g·L^− 1^)		
Haemorrhage-related reexploration	2.549 (1.132–5.738)	***0.02***
Septicaemia	1.293 (1.145–1.459)	***0.001***
Mean ICU stay (hours)	1.778 (1.469–2.087)	***0.04***
In-hospital mortality	2.133 (1.019–3.259)	***0.03***
**Normal Levels** (≥35 g·L^−1^) **vs Severe deficit** (< 25 g·L^− 1^)		
Haemorrhage-related reexploration	2.849 (2.132–3.138)	***0.01***
Septicaemia	2.025 (1.805–2.103)	***< 0.001***
Mean ICU stay (hours)	2.045 (1.690–2.235)	***< 0.001***
In-hospital mortality	3.206 (2.693–5.458)	***< 0.001***
**Low deficit** (30–34.9 g·L^− 1^) **vs Mild deficit** (25–29.9 g·L^− 1^)		
Haemorrhage-related reexploration	1.240 (1.122–1.350)	***0.04***
**Low deficit** (30–34.9 g·L^− 1^) **vs Severe deficit** (< 25 g·L^− 1^)		
Haemorrhage-related reexploration	1.259 (0.094–0.715)	***0.009***
Septicaemia	1.035 (1.015–1.303)	***< 0.001***
Mean ICU stay (hours)	2.580 (2.080–3.043)	***0.011***
In-hospital mortality	1.257 (1.103–1.624)	***0.003***
**Mild deficit** (25–29.9 g·L^− 1^) **vs Severe deficit** (< 25 g·L^− 1^)		
Septicaemia	1.035 (1.015–1.303)	***< 0.001***
In-hospital mortality	1.244 (1.130–1.456)	***< 0.001***

We also identified an association between chronic renal insufficiency, past CS and longer CPB times with the presence of mild to severe degree of hypoalbuminaemia at 24 h after CS (Table 6; see Supplementary Table 3 for full model results).

**Table 6:** Multivariate analysis – dependent variable having albumin levels < 30 g∙L^− 1^ 24 h after cardiac surgery

	Odds ratio (95% CI)	***P***-value
Chronic renal insufficiency	1.316 (1.085–1.595)	***0.005***
Hemoglobin before surgery (g·dL^**− 1**^)	0.860 (0.633–1.088)	*0.21*
Past Cardiac surgery	1.229 (1.067–1.415)	***0.004***
Cardiopulmonary bypass time (> 100 min)	1.904 (1.902–2.128)	***< 0.001***

## Discussion

The most important finding of the current study is the association of postoperative hypoalbuminemia with worst outcomes after CS depending on its intensity, even with influence in long-term scenario. To the best of our knowledge this is the only prospective study addressing the role of postoperative SAL in most types of CS procedures under the effect of CPB, even those with moderate to high complexity [12].

We excluded patients with low preoperative SAL because this may reflect a poor nutritional status that influences our results [2]. Several studies used different preoperative values as markers, making it difficult to determine a specific point to correlate these levels and a repercussion over time [8–10]. Despite the different values considered among these studies, it has been shown that patients with low preoperative SAL have an increased risk of morbidities and mortality after surgery [5–11]. The need for reoperation due to bleeding has been shown to be over 32% and mortality about 36.2% in patients with low SAL [3]. Preoperative SAL< 25 g∙L^− 1^ has been associated with higher mortality risk (OR 2.0; 95% CI, 1.3–3.0; *P* = 0.002) and a higher reoperation for bleeding [4]. In addition, preoperative SAL< 30 g∙L^− 1^ has been related with a prolonged ICU and in-hospital stay and a higher mortality compared with patients presenting normal SAL, as an independent risk factor [21]. Over the years, albumin has been considered as a negative acute-phase protein and a marker of inflammation [1], but it has not been taken into consideration to predict worse outcomes and mortality as a postoperative factor in CS. Since 1988, when it was first reported an increased risk of complications and prolonged hospital stay in elderly patients who presented preoperative low SAL [22], many other studies have been performed afterwards analyzing preoperative SAL [8–10]. Some studies have been performed showing altered protein metabolism after CS procedures, such as CABG, but no one took into consideration postoperative levels [23].

Patients undergoing CS develop a certain degree of SIRS depending on the CPB duration required during the intervention and other factors, such as hypothermia and blood transfusions, which may ultimately lead to the use of vasopressor drugs [12, 13]. Under this inflammatory condition, vital organs, such as the liver or kidneys, can worsen their function due to cellular damage, an increased vasodilation and extracapillar filtration leading to a loss of albumin to the extravascular space [24]. Following to this process, an increased fractional synthesis of albumin appears, stimulated by a lower oncotic pressure [24]. As a result, it is not surprising that the majority of our patients developed some degree of postoperative lower SAL.

On the other hand, postoperative SAL and their implications have been studied in other clinical contexts such as sepsis and acute kidney injury (AKI) in CS. The occurrence of sepsis in patients with hypoalbuminemia has a close pathophysiological relationship because the correct functioning of the immune response system depends on the metabolic and nutritional status, and vice versa [25]. Higher postoperative SAL may reflect a preserved lean body mass, which is related with nutritional reserve and a more efficient metabolic state, leading to a better inflammatory and immune response to surgery [2, 25]. On the other hand, sepsis is an important risk factor for mortality after CS, which produces a sepsis-induced cardiac dysfunction per se and preoperative hypoalbuminemia, has been shown to increase the risk for infection in CS [11].

Postoperative SAL may be useful as a predictive tool because is affected by several factors related with fluid and metabolic status [12]. Older age and smaller BMI are considered risk factors for bleeding complications, which are also related to higher rates of poor nutritional status [10, 11]. Indeed, in abdominal major surgeries, albumin drop is related to bleeding and major inflammatory response [26]. This may offer a possible explanation for the association of higher rates of sepsis and bleeding related complications, as well as the higher mortality, with lower SAL subgroups in our population.

We identified an association between chronic renal insufficiency, past CS and longer CPB times, with the presence of mild to severe degree of hypoalbuminaemia at 24 h after CS. A high proportion of patients with the need of a new CS suffer from heart failure, condition which severity and prognosis is closely associated with the presence of chronic inflammation and certain degree of malnutrition [27]. Chronic renal insufficiency is also associated with protein-energy wasting that leads to a similar clinical scenario [28]. Both clinical conditions, especially in the presence of chronic renal insufficiency, may be enhanced by SIRS caused by longer CPB times [28–30]. In consequence, underlying causes of hypoalbuminemia may also influence worst outcomes in SAL subgroups.

Administration of exogenous albumin in some critical scenarios has proved to be a controversial measure across different studies. In the case of sepsis and/or septic shock, the latest researches state there is no influence over short-term or long-term mortality and that its use may provide a certain hemodynamic improvement with no effect in survival rates [31]. On the contrary, a study proved that the use of exogenous albumin has a protective effect over the onset of AKI in patients with SAL < 40 g·L^− 1^ who underwent CS [32]. These various range of results prove the need to further analyze and research the use and determination of albumin in critical care areas.

There are certain limitations to this study, such as being a single-center long-term observational study, increasing the risk of losing track in the follow-up of some patients and the uncertainty over the causes of death in some cases. We described our fluid resuscitation protocol in detail but the exact amount of fluid challenge that could have influence postoperative SAL has not been provided. On the other hand, it presents several strengths. First, it is a prospective study in a large tertiary referral hospital, with a high level of complexity and a variety of all types of CS. In addition, we showed a large sample size that was further analyzed with systematic risk assessment, using preoperative and postoperative scores. Our study showed the importance of a proper stratification in patients undergoing CS. In addition, we have shown the risk profile of our CS population that may allow future comparisons with other series of CS patients, which is of great importance since the widespread use and importance of risk score stratification [15, 33]. We think it would have been interesting to check SAL several times after CS in order to provide better understanding about their behavior. However, this is beyond the scope and the aims of the present research.

## Conclusions

In summary, our study showed that the occurrence and the degree of hypoalbuminemia in the postoperative of CS is frequent and it may be associated with the development of several complications (especially septic or bleeding-related) and worst outcomes, even in long-term survival. Nutritional and inflammatory factors may be associated with the development of postoperative hypoalbuminemia. Postoperative SAL and factors associated with the development of mild to severe postoperative hypoalbuminemia may serve to early identify patients at risk of worst outcomes, which may ultimately help to intensity their monitoring and care in order to improve their status.

## Supplementary information

**Additional file 1.** Supplementary Figure 1 (consort diagram of the inclusion/ exclusion criteria), Supplementary Tables 1, 2 & 3 (correspond to full model results of Tables 2, 5 & 6), Ethics approval, Study protocol and collected data.

## Data Availability

Data has been provided in detail throughout the manuscript. The datasets used and/or analyzed during the current study are available from the corresponding author on reasonable request.

## Abbreviations

SAL: Serum albumin levels
CS: Cardiac Surgery
ICU: Intensive Care Unit
CPB: Cardio-pulmonary by pass
ACC: Aortic cross clamping
SIRS: Systemic inflammatory response syndrome
EuroSCORE: European System for Cardiac Operative Risk Evaluation
APACHE: Acute Physiology and Chronic Health Evaluation
SAPS: Simplified Acute Physiology Score
AMI: Acute Myocardial Infarction
MAP: Mean arterial pressure
AKI: Acute kidney injury
