# Supplementary material for: The influence of postoperative albumin levels on the outcome of cardiac surgery
Source: J Cardiothorac Surg. 2020 May 11;15:78. doi: 10.1186/s13019-020-01133-y (PMC7216430; doi:10.1186/s13019-020-01133-y)
Supplement: Supplementary file 1 — Additional file 1. Supplementary Figure 1 (consort diagram of the inclusion/ exclusion criteria), Supplementary Tables 1, 2 & 3 (correspond to full model results of Tables 2, 5 & 6), Ethics approval, Study protocol and collected data. [file 13019_2020_1133_MOESM1_ESM.docx]

***Supplementary Material:***

1. **Supplementary Figure 1.**
2. **Full Model results. Supplementary Tables 1, 2 & 3.**
3. **Ethics approval.**
4. **Study protocol.**
5. **Collected data.**

___________________________________________________________________________

**Supplementary Figure 1. Consort diagram of the inclusion/ exclusion criteria.**

**Supplementary Table 1.** Full Model results of multivariate analysis of Table 2– variables associated with in-hospital and long-term mortality.

| **Dependent variable in-hospital mortality** | | | | |
| --- | --- | --- | --- | --- |
| Steps | Variables included in the different final models | Wald | Odds ratio  (95% Confidence Interval) | *P*-value |
| 1 | Age (years) | 15.159 | 1.047 (1.023-1.071) | ***<0.001*** |
|  | CPB time (>100min) | 11.303 | 1.006 (1.002-1.009) | ***0.001*** |
|  | Hypertension | 1.044 | 0.913 (0.847-1.209) | *0.286* |
|  | Diabetes Mellitus | 1.244 | 0.980 (0.829-1.464) | *0.621* |
|  | COPD | 1.786 | 0.819 (0.727-1.273) | *0.375* |
|  | Creatinine before CS (μmol·l^-1^) | 2.117 | 1.001 (1.000-1.003) | *0.146* |
|  | Recent myocardial infarction | 0.453 | 0.851 (0.532-1.361) | *0.501* |
|  | Albumin 24h after surgery (g·L^-1^) | 7.059 | 0.944 (0.905-0.985) | ***0.008*** |
| 3 | Age (years) | 15.338 | 1.047 (1.023-1.071) | ***<0.001*** |
|  | CPB time (>100min) | 11.068 | 1.006 (1.002-1.009) | ***0.001*** |
|  | Hypertension | 1.126 | 0.807 (0.544-1.199) | *0.289* |
|  | COPD | 0.699 | 0.829 (0.734-1.287) | *0.403* |
|  | Creatinine before CS (μmol·l^-1^) | 2.153 | 1.001 (1.000-1.003) | *0.142* |
|  | Albumin 24h after surgery (g·L^-1^) | 7.472 | 0.943 (0.904-0.984) | ***0.006*** |
| 5 | Age (years) | 17.691 | 1.050 (1.027-1.075) | ***<0.001*** |
|  | CPB time (>100min) | 11.478 | 1.007 (1.003-1.010) | ***0.001*** |
|  | Creatinine before CS (μmol·l^-1^) | 2.761 | 1.002 (1.000-1.003) | *0.097* |
|  | Albumin 24h after surgery (g·L^-1^) | 7.295 | 0.844 (0.805-0.844) | ***0.007*** |

CPB: Cardiopulmonary bypass time; COPD: Chronic Obstructive Pulmonary Disease; CS: cardiac surgery.

| **Dependent variable long-term mortality** | | | | |
| --- | --- | --- | --- | --- |
| Steps | Variables included in the different final models | Wald | Odds ratio  (95% Confidence Interval) | *P*-value |
| 1 | Age (years) | 82.150 | 1.062 (1.049-1.077) | ***<0.001*** |
|  | CBP time (>100min) | 11.545 | 1.004 (1.002-1.006) | ***0.001*** |
|  | Hypertension | 0.020 | 0.984 (0.784-1.235) | *0.889* |
|  | Diabetes Mellitus | 10.223 | 1.590 (1.427-1.816) | ***0.001*** |
|  | COPD | 8.392 | 1.679 (1.527-1.822) | ***0.004*** |
|  | Creatinine before CS (μmol·l^-1^) | 8.205 | 1.002 (1.001-1.003) | ***<0.001*** |
|  | Recent myocardial infarction | 0.515 | 0.881 (0.624-1.244) | *0.473* |
|  | Dilated cardiomyopathy | 9.539 | 1.442 (1.143-1.819) | ***0.002*** |
|  | Treatment with statins before CS | 8.250 | 1.382 (1.118-1.724) | ***0.04*** |
|  | Albumin 24h after surgery (g·L^-1^) | 16.418 | 0.947 (0.922-0.972) | ***<0.001*** |
|  | Chest drainage first 12h after CS | 0.412 | 1.003 (0.899-1.005) | *0.783* |
|  | Acute Renal Failure | 12.526 | 1.823 (1.342-3.253) | ***<0.001*** |
|  | Low Cardiac Output Syndrome | 10.253 | 1.250 (1.126-1.865) | ***0.003*** |
|  | Septicaemia | 8.269 | 1.125 (1.018-1.696) | ***0.02*** |
| 3 | Age (years) | 82.166 | 1.062 (1.049-1.077) | ***<0.001*** |
|  | CBP time (>100min) | 11.736 | 1.004 (1.002-1.006) | ***0.001*** |
|  | Diabetes Mellitus | 7.281 | 1.428 (1.128-1.590) | ***0.021*** |
|  | COPD | 2.699 | 0.970 (0.789-1.187) | *0.103* |
|  | Creatinine before CS (μmol·l^-1^) | 5.230 | 1.002 (1.001-1.003) | ***0.012*** |
|  | Dilated cardiomyopathy | 9.513 | 1.442 (1.143-1.819) | ***0.002*** |
|  | Treatment with statins before CS | 0.517 | 0.982 (0.882-1.328) | *0.472* |
|  | Albumin 24h after surgery (g·L^-1^) | 16.635 | 0.947 (0.922-0.972) | ***<0.001*** |
|  | Acute Renal Failure | 14.335 | 1.723 (1.242-2.833) | ***<0.001*** |
|  | Low Cardiac Output Syndrome | 8.924 | 1.350 (1.126-2.625) | ***0.004*** |
|  | Septicaemia | 5.341 | 3.013 (1.182-7.680) | ***0.021*** |
| 6 | Age | 84.198 | 1.063 (1.049-1.076) | ***<0.001*** |
|  | CPB time (>100min) | 11.732 | 1.004 (1.002-1.006) | ***0.001*** |
|  | Diabetes Mellitus | 1.369 | 1.320 (0.928-1.790) | *0.23* |
|  | Dilated cardiomyopathy | 9.558 | 1.435 (1.139-1.810) | ***0.002*** |
|  | Albumin 24h after surgery (g·L^-1^) | 16.639 | 0.846 (0.821-0.871) | ***<0.001*** |
|  | Acute Renal Failure | 12.566 | 2.523 (1.395-2.933) | ***<0.001*** |
|  | Low Cardiac Output Syndrome | 9.364 | 1.489 (1.276-1.656) | ***0.006*** |
|  | Septicaemia | 5.427 | 1.125 (1.018-1.696) | ***0.02*** |

CPB: Cardiopulmonary bypass time; COPD: Chronic Obstructive Pulmonary Disease; CS: cardiac surgery.

**Supplementary Table 2.** Full Model results of multivariate analysis of Table 5. Differences between subgroups of patients with different albumin levels 24h after cardiac surgery.

| Comparison of subgroups with **Normal Levels** (≥35g·L^-1^) ***vs* Low deficit** (30-34.9g·L^-1^) | | | |
| --- | --- | --- | --- |
|  | Wald | Odds ratio (95% Confidence Interval) | *P*-value |
| Acute renal failure | 1.253 | 1.125 (0.950-1.894) | *0.15* |
| Haemorrhage-related reexploration | 0.035 | 1.167 (0.230-5.934) | *0.85* |
| Septicaemia | 2.331 | 1.743 (0.891-3.674) | *0.075* |
| Mean ICU stay (hours) | 0.237 | 1.005 (0.984-1.027) | *0.62* |
| In-hospital mortality | 7.854 | 1.018 (1.002-1.034) | ***0.02*** |

| Comparison of subgroups with **Normal Levels** (≥35g·L^-1^) ***vs* Mild deficit** (25-29.9g·L^-1^) | | | |
| --- | --- | --- | --- |
|  | Wald | Odds ratio (95% Confidence Interval) | *P*-value |
| Acute renal failure | 1.623 | 1.125 (0.750-1.894) | *0.15* |
| Haemorrhage-related reexploration | 6.237 | 2.549 (1.132-5.738) | ***0.02*** |
| Septicaemia | 9.527 | 1.293 (1.145-1.459) | ***0.001*** |
| Mean ICU stay (hours) | 5.428 | 1.778 (1.469-2.087) | ***0.04*** |
| In-hospital mortality | 6.724 | 2.133 (1.019-3.259) | ***0.03*** |

| Comparison of subgroups with **Normal Levels** (≥35g·L^-1^) ***vs* Severe deficit** (<25g·L^-1^) | | | |
| --- | --- | --- | --- |
|  | Wald | Odds ratio (95% Confidence Interval) | *P*-value |
| Ventilation time (hours) | 0.890 | 1.209 (0.902-1.470) | *0.12* |
| Acute renal failure | 1.053 | 1.149 (0.932-1.258) | *0.23* |
| Haemorrhage-related reexploration | 7.235 | 2.849 (2.132-3.138) | ***0.01*** |
| Septicaemia | 8.985 | 2.025 (1.805-2.103) | ***<0.001*** |
| Mean ICU stay (hours) | 6.233 | 2.045 (1.690-2.235) | ***<0.001*** |
| In-hospital mortality | 10.954 | 3.206 (2.693-5.458) | ***<0.001*** |

| Comparison of subgroups with  **Low deficit** (30-34.9g·L^-1^) ***vs* Mild deficit** (25-29.9g·L^-1^) | | | |
| --- | --- | --- | --- |
|  | Wald | Odds ratio (95% Confidence Interval) | *P*-value |
| Acute renal failure | 1.454 | 1.049 (0.989-1.150) | *0.43* |
| Haemorrhage-related reexploration | 4.550 | 1.240 (1.122-1.350) | ***0.04*** |
| Septicaemia | 1.286 | 1.025 (0.805-1.102) | *0.28* |
| Mean ICU stay (hours) | 1.085 | 1.045 (0.890-1.221) | *0.54* |
| In-hospital mortality | 1.203 | 1.253 (0.997-1.758) | *0.15* |

| Comparison of subgroups with  **Low deficit** (30-34.9g·L^-1^) ***vs* Severe deficit** (<25g·L^-1^) | | | |
| --- | --- | --- | --- |
|  | Wald | Odds ratio (95% Confidence Interval) | *P*-value |
| Acute renal failure | 0.454 | 1.039 (0.789-1.232) | *0.64* |
| Haemorrhage-related reexploration | 5.629 | 1.259 (0.094 - 0.715) | ***0.009*** |
| Septicaemia | 5.325 | 1.035 (1.015 - 1.303) | ***<0.001*** |
| Mean ICU stay (hours) | 4.989 | 2.580 (2.080 - 3.043) | ***0.011*** |
| In-hospital mortality | 7.945 | 1.257 (1.103 - 1.624) | ***0.003*** |

| Comparison of subgroups with  **Mild deficit** (25-29.9g·L^-1^) ***vs* Severe deficit** (<25g·L^-1^) | | | |
| --- | --- | --- | --- |
|  | Wald | Odds ratio (95% Confidence Interval) | *P*-value |
| Acute renal failure | 0.854 | 1.049 (0.989-1.150) | *0.70* |
| Haemorrhage-related reexploration | 1.950 | 1.122 (0.950-1.245) | *0.45* |
| Septicaemia | 4.750 | 1.035 (1.015 - 1.303) | ***<0.001*** |
| Mean ICU stay (hours) | 0.894 | 1.145 (0.990-1.320) | *0.64* |
| In-hospital mortality | 1.453 | 1.244 (1.130 - 1.456) | ***<0.001*** |

**Supplementary Table 3.** Full Model results of multivariate analysis of Table 6– dependent variable having albumin levels <30g∙L^-1^ 24h after cardiac surgery.

| Variables included | Wald | Odds ratio (95% Confidence Interval) | *P*-value |
| --- | --- | --- | --- |
| Age | 1.973 | 1.287 (0.983-1.991) | *0.54* |
| Hypertension | 0.179 | 1.020 (0.932-1.116) | *0.67* |
| Hemoglobin before surgery (g·dL^-1^) | 0.794 | 0.860 (0.633-1.088) | *0.21* |
| Chronic renal insufficiency | 7.802 | 1.316 (1.085-1.595) | ***0.005*** |
| Treatment with statins before CS | 1.238 | 1.053 (0.962-1.152) | 0.266 |
| Recent myocardial infarction | 0.420 | 1.011 (0.866-1.181) | 0.886 |
| Bypass surgery | 1.077 | 1.023 (0.869-1.204) | *0.782* |
| Cardiopulmonary bypass time (>100min) | 15.834 | 1.904 (1.902-2.128) | ***<0.001*** |
| Past Cardiac surgery | 8.132 | 1.229 (1.067-1.415) | ***0.004*** |

**Ethics approval: *This is a translation of the original document***

**REPORT OF THE ETHICS COMMITTEE AND CLINICAL RESEARCH ABOUT RESEARCH PROJECTS**

The Ethics Committee and Clinical Research of the Hospital Universitario de Bellvite, to date of 8^th^ March 2007, after examining all the documents about the research project with reference 38/07 entitled:

**“Observational Study of clinical variables after cardiac surgery and their influence in the evolution of the patients”**

Presented as main applicant by Dr. J. Ventura Farré from the Intensive Care Department in our hospital, has considered that there is not any ethical inconvenience for their performance and it has agreed to give definitive approval to this project, which implies a clinical database from the Intensive Care Department for their use in several observational studies based on the variables registered in this database.

Signed by Dr. Ramón Jodar Masanès

Ethics Committee and Clinical Research President

L’ Hospitalet de Llobregat, 8^th^ March 2007

**
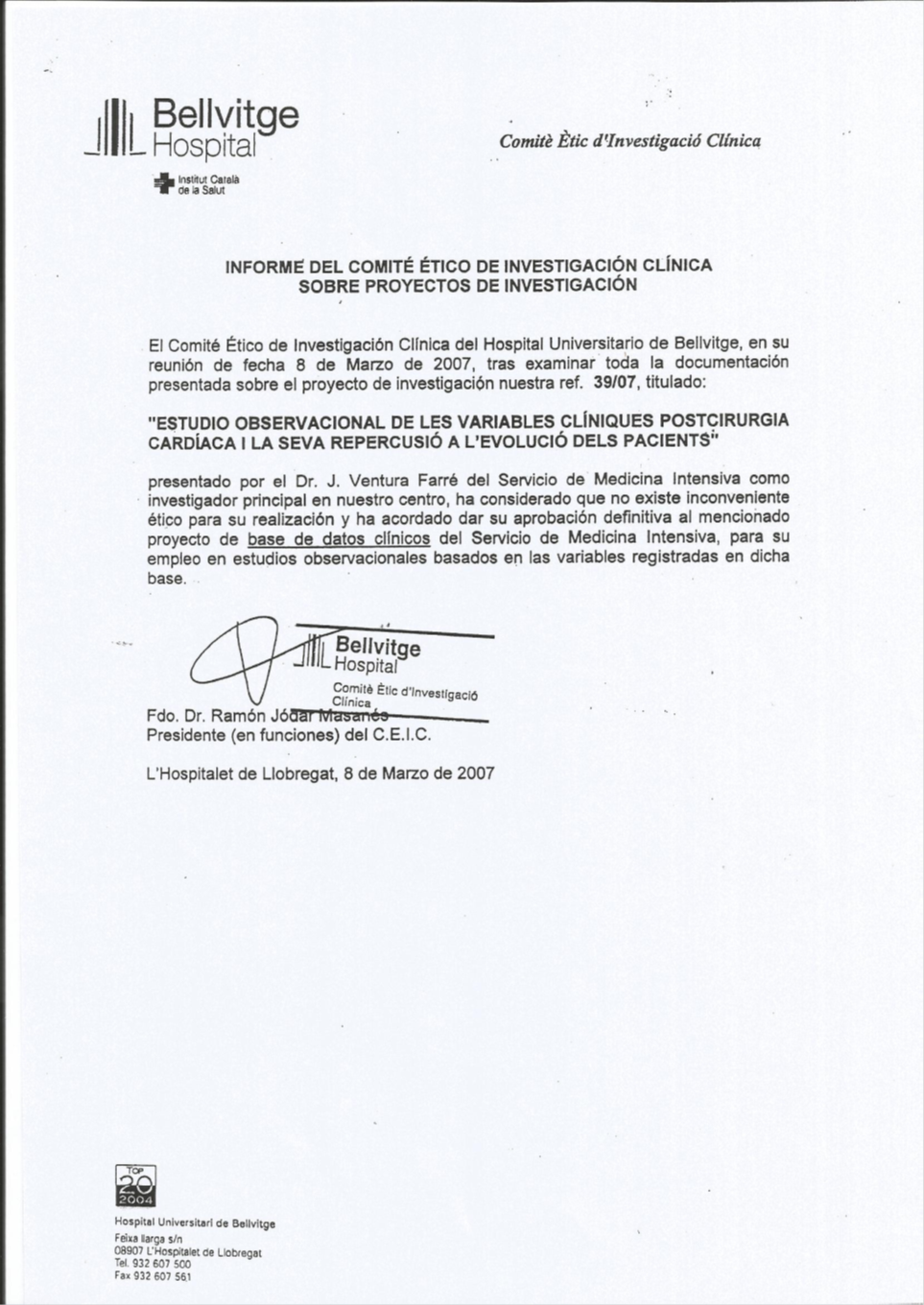
**

**Research Project:** Observational Study of clinical variables after cardiac surgery and their influence in the evolution of the patients.

Josep Lluís Ventura Farré^1,3^, Juan Carlos López Delgado^1,3^, Francisco Esteve Urbano^1,3^, David Rodríguez Castro^1^, Mª Lluïssa Carrió Cardona^1^, Elisabeth Farrero Bayarri^1^, Macarena Dastis Arias PhD^2^, Herminia Torrado MD^1^.

^1^ Intensive Care Department, Hospital Universitari de Bellvitge.

^2^ Clinical Laboratory Medicine Department, Hospital Universitari de Bellvitge.

^3^ IDIBELL (Institut d’Investigació Biomèdica Bellvitge; Biomedical Investigation Institute of Bellvitge).

Correspondence to: Juan Carlos Lopez-Delgado MD, PhD.

Consultant Physician. Hospital Universitari de Bellvitge, Intensive Care Department, IDIBELL (Institut d’Investigació Biomèdica Bellvitge; Biomedical Investigation Institute of Bellvitge). C/Feixa Llarga s/n. 08907, L’Hospitalet de Llobregat, Barcelona, Spain. [juancarloslopezde@hotmail.com](mailto:juancarloslopezde@hotmail.com) / [jclopez@bellvitgehospital.cat](mailto:jclopez@bellvitgehospital.cat)

Tel.: +34 650506985. Fax.: +34 932607963.

**ABSTRACT**

**Objectives:** Hospital mortality and morbidities have been declining in patients undergoing cardiac surgery (CS) despite their progressive aging and increasing complexity. With the trend towards greater longevity, patients tend to have higher preoperative morbidities and chronic illnesses. As a result, postoperative care and preoperative evaluation for adequate selection, or even preoperative interventions, are crucial for patients’ outcomes. Postoperative variables and laboratory data are both frequently not used to evaluate patients at risk of worst outcomes and CS scores have failed to be useful when applied to certain subpopulations. Inflammatory response seems to be crucial for the outcome of patients who undergo CS. In addition, laboratory parameters may have a potential better cost-effectiveness profile. Finally, the survival of postoperative CS is an important quality parameter of the surgery itself. However, this has not been sufficiently studied, assuming an area of particular interest for clinical research by the multiple implications that this entails.

Our aim is to evaluate the following items: Impact and epidemiology of subpopulations in CS (cirrhosis, AKI and Chronic Lung Disease) from the short- and long-term point of view (readmissions, new onset of comorbidities,…etc); Usefulness of the different scores (ICU, CS and cardiology scores); Long-term and quality of life evaluation, especially in those who have a prolonged ICU length of stay; Inflammatory response through classical and new promising biomarkers and determine its associated threshold with poor prognosis after CS; and most important to establish a registry within the current clinical practice for future evaluation of CS activity and identify future areas of intervention in the field of CS.

Methodology: Prospective observational study with a multidisciplinary project group which involves different specialties related with CS. Laboratory analysis will be performed in our hospital.

**1. BACKGROUND AND RATIONALE FOR THE STUDY.**

Hospital mortality and morbidities have been declining in patients undergoing cardiac surgery (CS) despite their progressive aging and increasing complexity [1, 2]. With the trend towards greater longevity, patients tend to have higher preoperative morbidities and chronic illnesses. As a result, population undergoing CS is becoming more heterogeneous over time, and postoperative care and preoperative evaluation for adequate selection, or even preoperative interventions, are crucial for patients’ outcomes [3].

Despite the quality of surgery and postoperative care for CS in a hospital requires several tools to compare the results with other centers, this is a difficult exercise due to the differences and the great heterogeneity within the different populations between specialized centers. The European System for Cardiac Operative Risk Evaluation (EuroSCORE) and Parsonnet score are specific scores to estimate the risk in CS. However, its predictive power decreases in certain populations, reaching not be valid. They also ignore many intraoperative variables, such as time of cardiopulmonary bypass (CPB), and the major part of postoperative variables [2]. The development of local mortality scores based on the epidemiological characteristics and the evaluation of postoperative variables can improve the prediction of mortality in the short- and long-term scenarios [1-9]. Specific CS scores may be also inaccurate for prognosis, especially in the case of surgical or postoperative complications. The Intensive Care Unit (ICU) own scores (Acute Physiology and Chronic Health Evaluation (APACHE) II and III, Simplified Acute Physiology Score (SAPS) II and III, and Sequential Organ Failure Assessment (SOFA) among others) include also chronic diseases in their evaluation and they are a better reflection of outcomes in CS population in some studies [10]. On the other hand, they are not specific for CS and require 24 hours for calculate them. Cardiologists have also developed several risk scoring systems for use in the clinical management of cardiovascular risk [11]. Another important consideration is that even the best score based on clinical factors does not always perform accurately enough in individual patients. Some unexplainable variations among patients may influence the predicted risk. Although postoperative CS is performed mainly in ICUs, scores are rarely published in literature, preventing a real comparison of results from different hospitals. Finally, the survival of postoperative CS is an important quality parameter of the surgery itself. However, this has not been sufficiently studied, assuming an area of particular interest for clinical research by the multiple implications that this entails.

Some particular populations, such as Liver Cirrhosis (LC) or older patients are expected to increase within the CS population in the next years. Risk factors for non-alcoholic steatohepatitis are the same that cardiovascular disease and population is expected to be older in Western countries [12]. Thus, there is growing need for a specific approach based on population characteristics and subpopulations with a particular disease due to epidemiological changes and the emergence of a greater number of patients within those particular subpopulations. The identification of subpopulations has also a high interest from the epidemiological point of view for better adjustment and planning of healthcare resources. The same applies for postoperative variables. Preoperative factors, such as LC [4, 5], or postoperative factors, such as the development of Acute Kidney Injury (AKI) [6], have become a major risk factor for CS due to increased morbidity and mortality. All contributing factors, emphasizing postoperative risk factors, need to be evaluated, especially from the long-term point of view for better understanding of the postoperative pathophysiology of CS and for evaluating the results of CS itself.

Laboratory parameters serve as guidance within the postoperative course of CS. However the exact role of them is poorly understood and the major part of them rarely appear in the results of many studies. For example, some evidence suggest that the arterial partial pressure of O2 and the fraction of inspired oxygen (PaO2/FiO2) ratio (an indicator of the state of oxygenation) may reflect persistent pulmonary dysfunction that may influence the prognosis in CS [7,13]. However, PaO2/FiO2 is poorly studied in CS and rarely appears in the results of the studies. The same happens with arterial blood lactate (AL). A high concentration of AL is common after CS, being a marker of heart failure associated with increased morbidity and mortality [14-16]. Although a higher lactate during the postoperative period has been associated with increased mortality, lactate values as a prognostic tool have not been fully studied. At the same time, adult CS carries a substantial risk of postoperative complications (e.g., bleeding, infections, organ dysfunction, and death in some cases). The causes are poorly understood, but activation of the inflammatory cascade plays a key role in the pathogenesis of those complications [17]. Cardiac surgery, especially under CPB, triggers a complex inflammatory response, which can lead to varying degrees of ischemia-reperfusion injury or systemic inflammatory response syndrome (SIRS). Depending on the intensity of this response, SIRS may progress to clinically relevant organ dysfunction [18]. Several factors such as surgical trauma, contact of blood with non-endothelialized surfaces leading to activation of the coagulation/complement cascade, ischemia-reperfusion, endotoxemia, hypothermia and blood transfusion contribute to the development of SIRS. In consequence, a better prediction of higher SIRS response would be helpful to plan and/or develop strategies to attenuate SIRS. The gold standard for the diagnosis of SIRS is based on recommendations provided in 1992 by the American College of chest Physicians and Society of clinical care medicine consensus conference, although, the SIRS criteria alone appear to be insufficient.

The important advantage of using laboratory parameters lies in its low cost: this information is provided by the analysis that does not require additional expense.‬ Besides the information that these parameters may supply, these biomarkers may be evaluated as predictors of adverse outcomes after CS. Biochemical, coagulopathy, hematological and blood gas parameters are required for a correct patient status interpretation. Additionally, N-terminal pro-B-type natriuretic peptide (NT-ProBNP), C reactive protein (CRP), lactate and prealbumin and albumin as a nutritional status markers may be measured. In concordance with bibliography, these biological quantities have an interesting prognostic value after CS [14-16, 19, 20]. Nevertheless, due to the lack of consensus, it should be necessary to confirm this approach and, through multivariate analysis, to know which provides better prognosis value.

In summary, despite advances in CS doctors have few tools available to assess the immediate prognosis in the postoperative course, especially if we consider that the progressive change in the pattern of this population remains a challenge and unanswered question for physicians and surgeons. Consequently, the understanding of the real epidemiology must be addressed and evaluation of the preoperative comorbidities (or acquired after surgery, such as chronic renal failure resulting from AKI) and postoperative complications in our population must be addressed if we want to optimize post-surgical management and improve their outcome. Evidence comes mainly from several small studies especially in the case of particular subgroups; due to the lack of evidence from larger prospective pools of data, more studies are still needed for this purpose. In most cases, the identification of specific populations remains a challenge for improving outcome and an adequate care in them, especially if their associated illness is underdiagnosed in the perioperative scenario. If we were able to identify these patients, we will be able to develop strategies in the management and therapeutics of them, minimizing the negative influence of their particular disease. The improvement of the knowledge of pathophysiology in the immediate postoperative period may help to develop tools in making decisions and influence the survival of the patients who undergo CS. Undoubtedly, better prognostic accuracy implies an improvement in establishing the degree of resources by each patient. Indeed more accurate long-term prognosis has also obvious and important advantages, both from the clinical and social point of view.

**2. HYPOTHESIS AND PROJECT OBJECTIVES.**

The following areas of research in Cardiac Surgery (CS) are not clearly elucidated: the impact and the epidemiology of subpopulations in CS and their resource consumption; usefulness and impact as prognosis tools of the different scores (ICU and CS scores among others); the quality of life and long-term outcomes of those patients who undergo CS, especially those who have a prolonged ICU length of stay; the role of current biochemical and haematological parameters and their associated threshold with poor prognosis after CS, especially in the diagnosis of SIRS, with postoperative complications.

In summary, the study will focus on the following items. Each item will mainly correspond to different sub-studies:

- Evaluate the impact and the epidemiology of subpopulations in our CS population, emphasizing preoperative and postoperative liver dysfunction, AKI and Chronic Lung Disease, especially tending into account their impact from the long-term point of view (readmissions, new onset of comorbidities,…etc).
- Evaluate the usefulness and impact as prognosis tool of the different scores (ICU and CS scores).
- Evaluate those patients who have a prolonged ICU length of stay (PICULOS) due to complications by means of the long-term evaluation, emphasizing also their quality of life.
- Evaluate inflammatory response through biochemical, haematological and coagulopathy parameters and determine its associated threshold with poor prognosis after CS.
- Evaluate utility of the research parameters in the diagnosis of SIRS.
- Establish a registry within the current clinical practice for future evaluation of CS activity and identify future areas of intervention in the field of CS. Data collection is expected to continue as part of clinical routine.

This study will be performed as a multidisciplinary project group (i.e., the co-workers on the current protocol), aiming the involvement of other co-workers involved in CS (i.e. anaesthesiology, cardiac surgeons and cardiologists) and the inclusion of other hospitals.

**3. RESOURCES AVAILABLE.**

This is an observational prospective study which would be conducted in a third level university affiliated hospital. We are experienced in large international randomized clinical trials, which would ensure successful accomplishment of the proposed study.

Our hospital is a referral in CS in the region, with a cardiac transplantation program. The expected increase of patients with comorbidities undergoing CS makes the present project a subject of special interest for the executive board of our hospital and public health in general.

In addition, there are few extra resources required for the study. Complementary tests needed to this project are mainly covered by the clinical routine of the hospital. All specialists are 24h at the hospital all days of the year. The follow-up (in our hospital) would be performed using the Catalan Health Central Registry (Registre Central de Persones Assegurades, RCA) and personal/ telephone contact with the patients.

**4. METHODS.**

This is a prospective observational study, aimed at collecting an adequate dataset on a large cohort of patients admitted to a postoperative cardiac surgery ICU.

INCLUSION CRITERIA:

• All patients who undergo all types of CS (with the exceptions described below).

EXCLUSION CRITERIA:

• Age < 16 years old.

• Only pericardial surgery.

SURGICAL PROCEDURES

Cardiac procedures in our hospital are performed using recommended surgical practice in all patients including median sternotomy, standard cardiopulmonary bypass (CPB) with moderate hypothermia (34ºC) and antegrade cardioplegia. A mean aortic pressure of > 60-70 mmHg is maintained during surgery. For revascularization we use the internal thoracic artery (or bilateral if possible) and saphenous vein grafts. Bypass graft flow are assessed for each graft by Doppler transit time flowmetry. Protamine is administered to reverse heparin according to standard practice. For CABG surgery, aspirin is routinely administered within the first 6 h (if indicated) after surgery following the local protocol. In all patients the decisions regarding postoperative ICU management are made by the attending ICU specialist, based on international guidelines, evidence-based medicine and local guidelines (e.g., antibiotic therapy).

DATA COLLECTION

Data on and during ICU admission would be extracted from the medical registry of each patient in real time using a standardized questionnaire and collected in a database for analysis purposes. This includes preoperative data (demographic data, comorbidities and treatment before surgery), operative data and postoperative variables usually measured on and during admission, which includes main outcomes. Definitions used for this study are based on the Society of Thoracic Surgeons’ national cardiac surgery database definitions. Recent myocardial infarction is defined as an AMI that required admission to the hospital during the last month before surgery or an AMI that did not allow discharge from the hospital before surgery or an AMI that happen in the last 90 days [21, 22]. Calculation of different prognosis scores for each patient would be done to identify best predictor for outcomes. In addition, necessary data would be registered in order to asses AKI risk scores in each patient. Long-term outcome includes readmissions (causes, characteristics…etc.), new onset of comorbidities (such as chronic renal failure), evaluation of quality of life after CS (especially in patients with PICULOS).

BIOCHEMICAL AND HAEMATOLOGICAL ANALYSIS

Sampling frequency would be the usual and has been evaluated by specialists of our hospital in order to avoid unnecessary sampling. Serum, plasma and whole blood will be collected for measurement of several parameters at baseline (preoperative), on UCI admission, at 6, 12, 24 and 48 h post UCI admission and at UCI discharge and hospital discharge (**Appendix 1**).

ETHICAL APPROVAL AND PATIENT’S CONSENT

We believe that informed patient consent will not be necessary, as this research is purely observational, the data collected are part of routine clinical care, and the data will be anonymized. Indeed, this research in performed without any conflict of interest and the aim has an implicit benefit for the patient and the society. However, we will notify the relevant ethics committee, in compliance with the local legislation and rules.

DATA STORAGE

The data is stored securely and all procedures regarding data management will comply with EU directive on data protection 95/46/EC. The database will be securely stored to avoid accidental or unauthorized disclosure or access. Access to the database will be granted to the investigators only, to perform the statistical analysis described in the attached plan. Investigators have the right to propose additional analysis of the collected data, subject to approval of the steering committee.

STATISTICAL ANALYSIS

The data to be collected are all collected as part of routine clinical care. Categorical variables will be described as proportions and will be compared using chi-square or Fishers exact test. Continuous variable will be described as mean and standard deviation if normally distributed or median and inter-quartile range if not normally distributed. Comparisons of continuous variables will be performed using one-way ANOVA or Mann-Whitney test as appropriate. Appropriate multivariable analysis, such as logistic regression model, will be performed to assess independent association between prognostic factors and outcomes, taking into account the hierarchical nature of the data. Significance will be set at p<0.05. A single final analysis is planned at the end of the study

Publication plan AND Deliverables

Data will be presented and disseminated in a timely manner. A writing committee will draft the scientific report(s) of this investigation. Specific funding is also requested to allow publication of data on an open access basis. The main deliverables will be scientific reports of preliminary findings for general and specialty journals and abstracts for presentation to national and international meetings. For each publication from the project, only contributors fulfilling the Vancouver criteria as outlined in the guidelines from the International Committee of Medical Journal Editors will be included as co-authors. Other contributors will be appropriately acknowledged. If data from the project are later used in separate publications, authorship would be based on the merits and contributions of each co-worker of the study and negotiated by the steering committee, even if some of the co-workers develop sub-studies based on ideas from full version of the present protocol.

CONFLICT OF INTEREST

The authors declare no conflict of interest.

**FUTURE.**

One of the future aims of this study is:

- Establish collaboration with other cardiac surgery centers in order to validate our results (National or European countries) and aiming at establishing collaborations.
- Develop interventions after identify the main problems of CS that serve for clinical trials, even in collaboration with the industry.
- Create a multidisciplinary approach of the patients who undergo CS in order to improve the results.

**REFERENCES.**

1. D'Ancona G, et al. Changing referral pattern in off-pump coronary artery bypass surgery: a strategy for improving surgical results. Heart Surg Forum 1999; 2: 246-9.

2. Roques F, Nashef SA, Michel P, Gauducheau E, de Vincentiis C, Baudet E, et al. Risk factors and outcome in European cardiac surgery: analysis of the EuroSCORE multinational database of 19030 patients. Eur J Cardiothorac Surg. 1999; 15: 816-22; discussion 822-3.

3. Fox AA, Nussmeier NA. Does gender influence the likelihood or types of complications following cardiac surgery? Semin Cardiothorac Vasc Anesth. 2004; 8:283-95.

4. Hayashida N, et al. Clinical outcome after cardiac operations in patients with cirrhosis. Ann Thorac Surg. 2004; 77: 500-5.

5. Suman A, et al. Predicting outcome after cardiac surgery in patients with cirrhosis: a comparison of Child-Pugh and MELD scores. Clin Gastroenterol Hepatol. 2004; 2:719-23.

6. Garwood S. Renal insufficiency after cardiac surgery. Semin Cardiothorac Vasc Anesth. 2004; 8:227-41.

7. Ng CS, et al. Pulmonary dysfunction after cardiac surgery. Chest. 2002;121:1269-77.

8. Gruberg L, et al. The impact of obesity on the short-term and long-term outcomes after percutaneous coronary intervention: the obesity paradox? J Am Coll Cardiol. 2002; 39:578-84.

9. Rivera-Fernández R, et al. Analysis of physiologic alterations in intensive care unit patients and their relationship with mortality. J Crit Care. 2007; 22:120-8

10. Geissler HJ, et al. Risk stratification in heart surgery: comparison of six score systems. Eur J Cardiothorac Surg. 2000; 17:400-6.

11. Conroy RM, et al. SCORE project group. Estimation of ten-year risk of fatal cardiovascular disease in Europe: the SCORE project. Eur Heart J. 2003; 24:987-1003.

12. McCullough AJ. Pathophysiology of nonalcoholic steatohepatitis. J Clin Gastroenterol. 2006; 40 Suppl 1:S17-29.

13. Williams TA, et al. Long-term survival from intensive care: a review. Intensive Care Med. 2005; 31:1306-15.

14. Demers P et al. Outcome with high blood lactate levels during cardiopulmonary bypass in adult cardiac operation. Ann Thorac Surg. 2000;70:2082-6.

15. Maillet JM, et al. Frequency, risk factors, and outcome of hyperlactatemia after cardiac surgery. Chest. 2003; 123: 1361-6.

16. Ranucci M, et al. Hyperlactatemia during cardiopulmonary bypass: determinants and impact on postoperative outcome. Crit Care. 2006; 10: R167.

17. Wan S, et al. Inflammatory response to cardiopulmonary bypass: Mechanisms involved and possible therapeutic strategies. Chest. 1997, 112: 676-692.

18. Hirai S. Systemic inflammatory response syndrome after cardiac surgery under cardiopulmonary bypass. Ann ThoracCardiovasc Surg. 2003, 9: 365-370.

19. Morimoto K, et al. Perioperative changes in plasma brain natriuretic peptide concentrations in patients undergoing cardiac surgery. Surg Today. 1998 ;28:23-9.

20. Gaspardone A, et al. Predictive value of C-reactive protein after successful coronary-artery stenting in patients with stable angina. Am J Cardiol. 1998; 82: 515-8.

21. Priebe HJ. Perioperative myocardial infarction--aetiology and prevention. Br J Anaesth. 2005; 95:3-19.

22. Eagle KA, et al. American College of Cardiology American Heart Association. ACC/AHA 2004 guideline update for coronary artery bypass graft surgery: a report of the American College of Cardiology/American Heart Association Task Force on Practice Guidelines (Committee to Update the 1999 Guidelines for Coronary Artery Bypass Graft Surgery). Circulation 2004, 110: e340-437.

**Appendix 1**

**Biochemical and haematological analysis. Sampling frequency**

|  | **Preoperative** | **On UCI admission** | **6 h** | **12 h** | **24 h** | **48 h** | **On UCI discharge** | **On hospital discharge** |
| --- | --- | --- | --- | --- | --- | --- | --- | --- |
| Glucose | x | x | x | x | x | x | x | x |
| Creatinine | x | x | x | x | x | x | x | x |
| Urea | x | x | x | x | x | x | x | x |
| Sodium | x | x | x | x | x | x | x | x |
| Potassium | x | x | x | x | x | x | x | x |
| Total bilirubin | x | x | x | x | x | x | x | x |
| ALT | x | x | x | x | x | x | x | x |
| Albumin | x |  |  |  | x | x | x | x |
| Cholesterol | x |  |  |  |  |  |  | x |
| LDL cholesterol | x |  |  |  |  |  |  | x |
| HDL cholesterol | x |  |  |  |  |  |  | x |
| Triglyceride | x |  |  |  |  |  |  | x |
| CRP | x | x |  |  | x | x | x | x |
| Homocysteine | x |  |  |  |  |  |  |  |
| NT-proBNP | x | x |  |  | x |  |  |  |
| Arterial lactate | x | x | x | x | x | x | x | X |
| TNT-hs | x | x | x | x | x | x | x | x |
| PT | x | x | x | x | x | x | x | x |
| Haemogram | x | x | x | x | x | x | x | x |
| ABG | x | x | x | x | x | x | x | x |

ALT: Alanine aminotrasferase, CRP: C reactive protein, NT-proBNP: N-terminal pro-B-type natriuretic peptide, TNT-hs: Troponin T high sensitive, PT: partial thromboplastin time, ABG: blood gas analysis.

**Collected data:**

**ID (Study Number): _____________**

**PREOP data:**

**Age: _____** years **Gender:** Male / Female **Weight: _____**cm **Height: _____**kg

**Hospital admission date: ____/____/____**

**Previous state:**

**Smoking:** Current smoker / <6moths / Never Alcohol: No / Yes

**Hypertension:** No / Yes **Diabetes:** No / Yes Dyslipidemia: No / Yes

**Previous Myocardial Infarction:** No / Yes **<90 days AMI:** No / Yes

**Complication from MI:** 1.No. 2.Ventricular Septum Rupture. 3.Mitral Insufficiency.

**Peripheral vascular disease:** No / Yes

**Thoracic aortic disease:** 1. No 2. Dissection. 3. Aneurysm.

**Cerebrovascular disease:** No / Yes **Poor mobility**: No / Yes

**Chronic cardiac insufficiency:** No / Yes

**NYHA class: I II III IV**

**Previous Atrial Fibrillation:** No / Yes **Sinus rhythm:** No / Yes

**Echocardiography: LVEF:_____**% **PAP:_____**mmHg

**LV Hypertrophy:** No / Yes LV Dilatation: No / Yes

**Pulmonary Disease:** No / Yes COPD: No / Yes

**Spirometry: FEV_1_:_____** FVC:______

On dialysis: No / Yes Last dialysis prior to surgery:______ Days

**Renal transplantation:** No / Yes

**Endocarditis:** No / Yes On Antibiotic therapy until surgery: No / Yes

**Previous Treatment:**

**Statins:** No / Yes  **Other Lipid lowering drug medication:** No / Yes

**Antidiabetics:** No / oral / insulin

**Diuretic:** No / Yes **Nitrate:** No / Yes

**β-blokers**: No / Yes **Calcium blocker:** No / Yes **ACE inhibitors** or **ARA II:** No / Yes

**Aspirin:** No / Yes

***Discontinued:*** 1.No. 2. Yes. _______ days ago.

**Clopidogrel** /**Ticagrelor** / **Briticagrelor**: No / Yes

***Discontinued:*** 1.No. 2. Yes. _______ days ago.

**Anticoagulation:** No / Yes

**Thrombin inhibitor / Factor X inhibitor / Warfarin / Heparin / LMWH**

***Discontinued:*** 1.No. 2. Yes. _______ days ago.

**Anti-arrhythmia:** No / Yes **Digitalis:** No / Yes

**Bronchodilators:** No / Yes **Steroids:** No / Yes

**Critical preoperative state:**

1. **Ventricular tachycardia or ventricular fibrillation.**
2. **Aborted sudden death or preoperative cardiac massage.**
3. **Preoperative mechanical ventilation before anaesthetic room.**
4. **Preoperative inotropes.**
5. **Preoperative IABP.**
6. **Preoperative acute renal failure (anuria or oliguria <10ml/hr).**
7. **Preoperative acute Cardiac Insufficiency.**
8. **NO.**

**OPERATIVE Data:**

**Previous Cardiac Surgery:** No / Yes **Date: ____ /____ /____**

**Degree of urgency:**

**1. Elective (Standard waiting list). 2. Urgent (Operation within 2 weeks).**

**3. Emergency (Operation within 24h).** **4. Salvage (Operation within 24h).**

**Operation Type:**

1. **CABG.**

- **Incomplete revascularization:**

No / Yes

- **Number of Bypasses:_____**

1. **Aortic valve replacement (AVR).**
2. **Mitral valve replacement:**

- **Ischemic mitral valve:** No / Yes

1. **Ascending aorta surgery:**

- **Aneurysm___________**
- **Dissection___________**

1. **Tricuspid valve surgery.**
2. **Atrial/ Ventricular septum defect repair.**
3. **Pericardiectomy.**
4. **Removal of cardiac tumors.**
5. **Other:____________.**

**CPB during surgery:** No / Yes

**CPB time: _____**min **ACC time: _____**min

**Total time on surgery:____**min

Biological valve (If applicable): No / Yes

**Standard CPB:** No / Yes **If No:**

- 1. **Antegrade cerebral perfusion (aortic arch surgery/ total circulatory arrest).**

**Total time on circulatory arrest: __________min**

- 1. **Distal aortic perfusion.**

***Other complications during surgery/ Comments:***

**POST-OPERATIVE Data:** **ICU Admission Date: ____ / ____ /_____**

***Transfusion requirements & bleeding complications:***

Pericardial tamponade: No / Yes

**Re-exploration:** No / Yes

**1. Bleeding. 2. Bleeding and tamponade. 3. Sternum loosening.**

**4. Circulatory Collapse.**

**5. Other:_________________________** Surgical cause for bleeding: No / Yes

| **Transfusion** | **Intraoperative** | **1^st^ PO day** | **Postoperative** |
| --- | --- | --- | --- |
| **Blood** (Units) |  |  |  |
| **Plasma** (Units) |  |  |  |
| **Platelets** (Units) |  |  |  |
| Tranexamic acid (g) |  |  |  |
| Other |  |  |  |
| **Lowest Hb (g∙dL^-1^)** |  |  |  |
| Lowest Platelets |  |  |  |
| Worst INR or PT |  |  |  |

|  | **3h** | **4h** | **12h** | **Total** |
| --- | --- | --- | --- | --- |
| **Drainage loss** (mL) |  |  |  |  |

***Hemodynamic*:**

**Perioperative AMI:** No / Yes Killip: I II III IV

**Postoperative arrhythmia:** 1. Atrial fibrillation or flutter. 2. Other.

***Abdominal complications:*** No / Yes

**1. GI bleeding. 2. Cholecystitis. 3. Pancreatitis. 4. Liver failure. 5. Mesenterial ischemia.**

**6. Ileus. 7. Other__________________**

| **Inotropic support** | **Pre-surgery** | **Surgery** | **Inotropic support for ICU transfer** | **After surgery** | **Total time on support** |
| --- | --- | --- | --- | --- | --- |
| **DBT^1^**(cc/h or µg·kg^-1^·min^-1^) |  |  | No / Yes |  | **hrs** |
| **DP^2^** (cc/h or µg·kg^-1^·min^-1^) |  |  |  |  | **hrs** |
| **NA^3^** (cc/h or µg·kg^-1^·min^-1^) |  |  |  |  | **hrs** |
| **Levosimendan*** (mg) |  |  |  |  | **NA** |
| **IABP^4^ support** | No / Yes | No / Yes |  | No / Yes | **hrs** |
| **Defibrillation (for VF^5^)** | No / Yes | No / Yes |  | No / Yes | **NA** |
| **ECMO^6^** | No / Yes | No / Yes |  | No / Yes | **days** |
| **Cardiac arrest** | No / Yes | No / Yes |  | No / Yes | **NA** |

**^1^Dobutamine, ^2^Dopamine, ^3^Noradrenaline, ^4^Intra-aortic Balloon Pump, ^5^Ventricular Fibrillation, ^6^ExtraCorporeal Membrane Oxygenation. * Simdax®.**

***Acute kidney Injury:***

**Max. serum Creatinine (during ICU admission): _______** µmol·L^-1^

Max. urea (during ICU admission): _______mmol·L^-1^

|  | **Surgery** | **First 24h** | **ICU admission** |
| --- | --- | --- | --- |
| **Fluid Balance** |  |  |  |
| **RRT^1^** | No / Yes | No / Yes | No / Yes |

**^1^ Renal Replacement Therapy**

***Neurological complications:*** No / Yes

1. **Stroke 2. TIA 3.Coma (Post-anoxia) 4.Delirium/Phycosis.**

***Ischemia of arm/leg:*** No / Yes

|  | **Pre-Surgery** | **ICU admission*** | **6h*** | **12h*** | **24** | **48h** | **ICU Discharge** | **1-week after CS** | **Hospital Discharge** |
| --- | --- | --- | --- | --- | --- | --- | --- | --- | --- |
| **Glucose** |  |  |  |  |  |  |  |  |  |
| **Creatinine** |  |  |  |  |  |  |  |  |  |
| **Urea** |  |  |  |  |  |  |  |  |  |
| **Na+** |  |  |  |  |  |  |  |  |  |
| **K+** |  |  |  |  |  |  |  |  |  |
| **Total Bilirrubin** |  |  |  |  |  |  |  |  |  |
| **ALT** |  |  |  |  |  |  |  |  |  |
| **AST** |  |  |  |  |  |  |  |  |  |
| **Alkaline phosphatase** |  |  |  |  |  |  |  |  |  |
| **Gamma -GT** |  |  |  |  |  |  |  |  |  |
| **Prealbumin** |  |  |  |  |  |  |  |  |  |
| **Proteins** |  |  |  |  |  |  |  |  |  |
| **Ca+** |  |  |  |  |  |  |  |  |  |
| **Albumin** |  |  |  |  |  |  |  |  |  |
| **Cholesterol** |  |  |  |  |  |  |  |  |  |
| **LDL** |  |  |  |  |  |  |  |  |  |
| **HDL** |  |  |  |  |  |  |  |  |  |
| **Triglycerides** |  |  |  |  |  |  |  |  |  |
| **C-Reactive Protein** |  |  |  |  |  |  |  |  |  |
| **Arterial Lactate*** |  |  |  |  |  |  |  |  |  |
| **Troponin*** |  |  |  |  |  |  |  |  |  |
| **PT** |  |  |  |  |  |  |  |  |  |
| **aPTT** |  |  |  |  |  |  |  |  |  |
| **Fibrinogen** |  |  |  |  |  |  |  |  |  |
| **Hemoglobin** |  |  |  |  |  |  |  |  |  |
| **Platelets** |  |  |  |  |  |  |  |  |  |
| **WBC** |  |  |  |  |  |  |  |  |  |
| **Lymphocytes** |  |  |  |  |  |  |  |  |  |
| **Arterial Blood Gas *** |  |  |  |  |  |  |  |  |  |
| **Venous Blood Gas*** |  |  |  |  |  |  |  |  |  |
| **urinary urea (24h U.O)** |  |  |  |  |  |  |  |  |  |
| **Immature platelet fraction** |  |  |  |  |  |  |  |  |  |

*** urgent samples.**

***Respiratory values:***

Postoperative intubation time:______hs

**Re-intubation:** No / Yes **Number of days/hs for reintubation:____**

**Tracheostomy:** No / Yes Tracheostomy date: ____ / ____ /_____

**ARDS** (*Immflamatory*)**:** No / Yes

NIV after extubation: No / Yes NIV failure: No / Yes Time on NIV: ____hrs

Max.Pressure support/IPAP needed NIV: _____

Max.PEEP/EPAP needed NIV: _____

Cause for use NIV/ Re-reintubation:

1. Acute Pulmonary Edema/ Heart failure

2. Other: ________________

**Total postoperative intubation time:** _____ hs Max.PEEP needed: _____

**Deep venous thrombosis:** No / Yes **Pulmonary embolism:** No / Yes

***Infectious complications:*** No / Yes

**Type of infection:**

1. **Pulmonary: Pneumonia / Tracheobronchitis**
2. **Urinary**
3. **Catheter related**

- **Venous: femoral / Jugular / Subclavian /** Humeral
- **Arterial: femoral / radial / Other**

1. **Wound infection: 1. Thorax. 2. Lower Limb. 3. Other.**
2. **Mediastinitis: re-exploration (drained): No / Yes**

Type of microorganism: ______________________

**Adequate antibiotic treatment:** No / Yes _________________

Inflammatory response infection-related:

1. Sepsis 2. Severe sepsis 3. Septic Shock 4. Multiorgan failure

|  | During ICU admission |
| --- | --- |
| Worst White Blood Cells count |  |
| Maximum serum Platelets |  |
| Maximum C-reactive protein (mg·L^-1^) |  |
| Maximum Arterial lactate |  |

***ICU Scores (calculated first 24hs after admission):***

SOFA:

APACHE II:

APACHE III:

SAPSII:

SAPS III:

***Outcome:***

**ICU discharge Date:** **____ / ____ /_____** **ICU Death:** No / Yes

**Hospital discharge Date: ____ / ____ /_____** **In-hospital** **Death:** No / Yes

**Discharge to: 1. Home. 2. Other hospital. 3. Rehab.**

**Cause of death:**

- - - 1. **Bleeding.**
      2. **Cardiac.**
      3. **Cerebral.**
      4. **Pulmonary.**
      5. **MOF.**
      6. **Other: __________________________**

*Follow-up:*

Follow-up death: No / Yes Death Date: ____ / ____ /_____

***Hemodynamic values:***

|  | **ICU admission** | **6h** | **12h** | **18h** | **24h** | **Day 2** | **Day 3** | **…** |
| --- | --- | --- | --- | --- | --- | --- | --- | --- |
| **Mean BP (mmHg)**  **(Systolic/ Diastolic)** |  |  |  |  |  |  |  |  |
| **Heart Rate** |  |  |  |  |  |  |  |  |
| **Arrhythmia?** | **Y/N** | **Y/N** | **Y/N** | **Y/N** | **Y/N** | **Y/N** | **Y/N** |  |
| **Central Venous Pressure (mmHg)** |  |  |  |  |  |  |  |  |
| **Left Atrial Pressure (mmHg)** |  |  |  |  |  |  |  |  |
| **Swan-Ganz®** | **ICU admission** | **6h** | **12h** | **18h** | **24h** | **Day 2** | **Day 3** | **…** |
| **Cardiac Index (L·min^-1^·m^-2^)** |  |  |  |  |  |  |  |  |
| **Mean Pulmonary BP (mmHg)**  **(Systolic/ Diastolic)** |  |  |  |  |  |  |  |  |
| **Pulmonary Artery Occlusion Pressure (mmHg)** |  |  |  |  |  |  |  |  |
| **Systemic Vascular Resistance Index (dynes-sec·cm^–5^·m^2^)** |  |  |  |  |  |  |  |  |
| **Pulmonary Vascular Resistance Index (dynes-sec·cm^–5^·m^2^)** |  |  |  |  |  |  |  |  |
| **ProAQT®** | **ICU admission** | **6h** | **12h** | **18h** | **24h** | **Day 2** | **Day 3** | **…** |
| **Cardiac Index (L·min^-1^·m^-2^)** | **ICU admission*** | **6h*** | **12h*** | **24** | **48h** | **ICU Discharge** | **1-week after CS** | **Hospital Discharge** |
| **Stroke Volume Variation (%)** |  |  |  |  |  |  |  |  |
